# Taxonomy and physiology of *Pseudoxanthomonas arseniciresistens* sp. nov., an arsenate and nitrate-reducing novel *gammaproteobacterium* from arsenic contaminated groundwater, India

**DOI:** 10.1371/journal.pone.0193718

**Published:** 2018-03-20

**Authors:** Balaram Mohapatra, Pinaki Sar, Sufia Khannam Kazy, Mrinal Kumar Maiti, Tulasi Satyanarayana

**Affiliations:** 1 Department of Biotechnology, Indian Institute of Technology Kharagpur, Kharagpur, West Bengal, India; 2 Department of Biotechnology, National Institute of Technology Durgapur, Durgapur, West Bengal, India; 3 Department of Microbiology, University of Delhi South Campus (UDSC), New Delhi, Delhi, India; University of Porto, PORTUGAL

## Abstract

Reductive transformation of toxic arsenic (As) species by As reducing bacteria (AsRB) is a key process in As-biogeochemical-cycling within the subsurface aquifer environment. In this study, we have characterized a Gram-stain-negative, non-spore-forming, rod-shaped As reducing bacterium designated KAs 5-3^T^, isolated from highly As-contaminated groundwater of India. Strain KAs 5-3^T^ displayed high 16S rRNA gene sequence similarity to the members of the genus *Pseudoxanthomonas*, with *P*. *mexicana* AMX 26B^T^ (99.25% similarity), *P*. *japonensis* 12-3^T^ (98.9 0%), *P*. *putridarboris* WD-12^T^ (98.02%), and *P*. *indica* P15^T^ (97.27%) as closest phylogenetic neighbours. DNA-DNA hybridization study unambiguously indicated that strain KAs 5-3^T^ represented a novel species that was separate from reference strains of *P*. *mexicana* AMX 26B^T^ (35.7%), *P*. *japonensis* 12-3^T^ (35.5%), *P*. *suwonensis* 4M1^T^ (35.5%), *P*. *wuyuanensis* XC21-2^T^ (35.0%), *P*. *indica* P15^T^ (32.5%), *P*. *daejeonensis* TR6-08^T^ (32.0%), and *P*. *putridarboris* WD12^T^ (22.1%). The DNA G+C content of strain KAs 5-3^T^ was 64.9 mol %. The predominant fatty acids were C_15:0_ (37.4%), C_16:0_ iso (12.6%), C_17:1_ iso ω9c (10.5%), C_15:0_ anteiso (9.5%), C_11:0_ iso 3-OH (8.5%), and C_16:1_ ω7c/ C_16:1_ ω6c (7.5%). The major polar lipids were diphosphatidylglycerol, phosphatidyldimethylethanolamine, phosphatidylcholine, and two unknown phospholipids (PL1, PL2). Ubiquinone 8 (Q8) was the predominant respiratory quinone and spermidine was the major polyamine of the strain KAs 5-3^T^. Cells of strain KAs 5-3^T^ showed the ability to use O_2_, As^5+^, NO_3_^-^, NO_2_^-^, and Fe^3+^ as terminal electron acceptors as well as to reduce As^5+^ through the cytosolic process under aerobic incubations. Genes encoding arsenate reductase (*ars*C) for As-detoxification, nitrate- and nitrite reductase (*nar*G and *nir*S) for denitrification were detected in the strain KAs 5-3^T^. Based on taxonomic and physiological data, strain KAs 5-3^T^ is described as a new representative member of the genus *Pseudoxanthomonas*, for which the name *Pseudoxanthomonas arseniciresistens* sp. nov. is proposed. The type strain is KAs 5-3^T^ (= LMG 29169^T^ = MTCC 12116^T^ = MCC 3121^T^).

## Introduction

Taxonomic hierarchy of the genus *Pseudoxanthomonas* denotes its affiliation to the class *Gammaproteobacteria*, family *Xanthomonadaceae* of phylum *Proteobacteria*. Members of the genera *Xanthomonas*, *Xyllela*, and *Stenotrophomonas* are found to be the nearest phylogenetic neighbours of *Pseudoxanthomonas* [1]. Finkmann *et al*. [2] reported the first validly described species of *Pseudoxanthomonas*, *P*. *broegbernensis* isolated from an experimental biofilter. The taxon has been subsequently emended by Thierry *et al*. [3] and Lee *et al*. [4]. Members of this genus were described as Gram-stain-negative, non-spore forming rods, with iso C_15:0_ and anteiso C_15:0_ as major fatty acids, ubiquinone (Q8) as major respiratory quinone and capable of performing strict respiratory metabolism with O_2_ as preferred terminal electron acceptor [3]. The genus can be well differentiated from the two other related members *Xanthomonas* and *Stenotrophomonas* by the absence of fatty acid C_13:0_ 3-OH and from genus *Xylella* by the presence of branched-chain fatty acids (as described in the Bergey’s Manual of Systematic Bacteriology, 2^nd^ edition, Volume II, The *Proteobacteria* [4]). At the time of writing this manuscript, 17 validly described and two non-validly described (but effectively published) type species of the genus *Pseudoxanthomonas* were reported from varied environments [2–17]. The non-validly described members (but effectively published): *P*. *kaohsiungensis* and *P*. *gei* are isolated from an oil-polluted site and plant stem respectively [18, 19]. The members of this genus are ecologically important due to their ability to reduce both nitrite and nitrate; degrade a variety of hydrocarbons (including benzene, toluene, ethyl-benzene and *o*-, *m*-, *p*- xylene) [20–22]. Recently, the presence of *Pseudoxanthomonas* and other members of *Xanthomonadaceae* have been reported for As-contaminated groundwater of alluvial aquifers in West Bengal and Bangladesh [23–26]. However, neither the taxonomic identity of these strains nor their eco-physiology towards As-transformation has been adequately studied. As a result, the role of such organisms in biogeochemical-cycling of As in contaminated groundwater remained highly unexplored.

The present study was therefore undertaken to investigate the taxonomic and eco-physiological properties of an As-resistant and -reducing *Pseudoxanthomonas* strain previously isolated from As rich groundwater of West Bengal [23]. A polyphasic taxonomic approach was undertaken to characterize and delineate the taxonomic position of the strain KAs 5-3^T^. This strain was found to possess abilities for anaerobic As reduction and hydrocarbons utilization as well as several other traits potentially important for surviving in highly As-contaminated oligotrophic aquifer environment. To the best of our knowledge, till date no *Pseudoxanthomonas* type strain has been characterized from As-contaminated groundwater and capable of reducing toxic As^5+^ while assimilating hydrocarbons.

## Materials and methods

### Bacterial strains and culture conditions

The strain KAs 5-3^T^ (LMG 29169^T^ = MTCC 12116^T^ = MCC 3121^T^) was originally isolated from an As-contaminated groundwater (total As of 500 μg/L, salinity of 0.4 parts per thousand) of West Bengal [23]. Type strains of *Pseudoxanthomonas* (*P*. *mexicana* AMX 26B^T^, *P*. *japonensis* 12-3^T^, *P*. *indica* P15^T^, *P*. *suwonensis* 4M1^T^, *P*. *wuyuanensis* XC21-2^T^, *P*. *putridarboris* WD12^T^, and *P*. *daejeonensis* TR6-08^T^) were obtained from various culture collections [Japan Collection of Microorganisms (JCM, Japan), Microbial Type Culture Collection (MTCC, India), Korean Type Culture Collection (KCTC), and Korean Agricultural Culture Collection (KACC, Korea)] and used as reference organisms in various experiments. Strain KAs 5-3^T^ and the reference type strains were routinely sub-cultured and maintained on Luria-Bertani broth (g L^-1^; Casein enzymic hydrolysate, 10.0; yeast extract, 5.0; NaCl, 10.0; pH adjusted to 7.5) or minimal salt medium (MSM) (g L^-1^; Tris buffer, 6.0; NaCl, 5.0; KCl, 1.52; NH_4_Cl, 1.04; Na_2_SO_4_, 0.4; MgCl_2_.6H_2_O, 0.2; CaCl_2_.2H_2_O, 0.03; K_2_HPO_4_, 0.01; KH_2_PO_4_, 0.01, pH adjusted to 7.0). For MSM, either glucose (10 mM, v/v) or yeast extract (2.0%, v/v) was used as carbon source, as appropriate.

### 16S rRNA gene phylogeny and multi locus sequence typing

**Fig 1:**
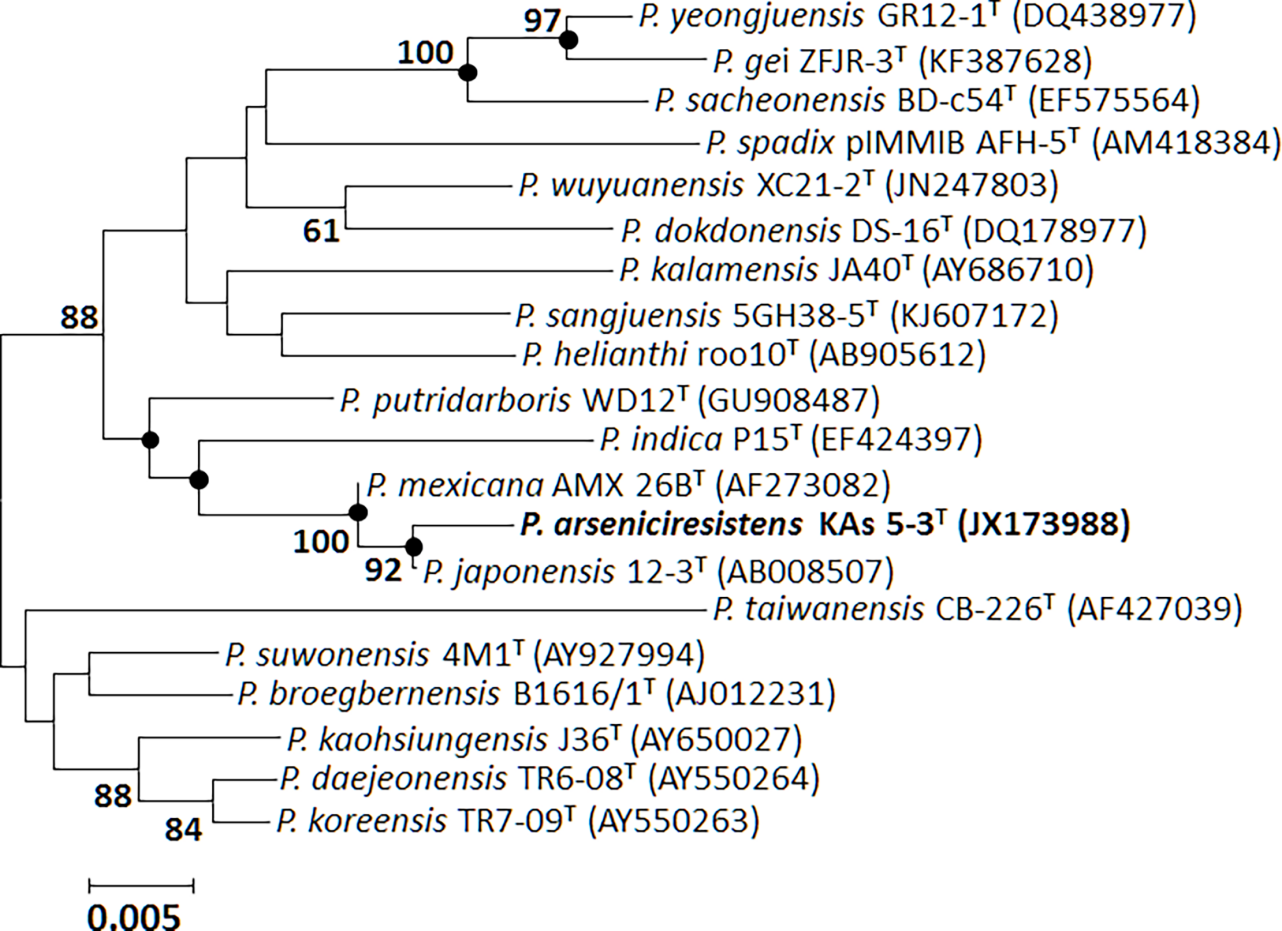
Neighbour-joining phylogenetic tree based on 16S rRNA gene sequence of KAs 5-3^T^ with type strains of the genus *Pseudoxanthomonas*. Bootstraps (1000 replications) of above 60% are shown at each branch points. A total of 1492 positions involving 20 nucleotide sequences were considered in the final dataset for construction of the tree. Filled circles indicate that the corresponding nodes were also recovered in trees generated with maximum-likelihood and minimum-evolution algorithm. Bar 0.005 indicates 0.5% substitution.

Nearly complete stretch of 16S rRNA gene was PCR amplified using 27F/1492R primers (Table A in S1 File); individual sequences were edited and assembled by BioEdit version 7.1.11 [27], subjected to similarity search in NCBI BLAST [28], RDP II [29], and against validly described members in the EzBioCloud database (http://www.ezbiocloud.net/eztaxon; [30]). Multiple alignments with 16S rRNA gene sequences of *Pseudoxanthomonas* type strains were performed using the CLUSTAL W package of the MEGA software version 7.0 [31]. All ambiguous positions were removed for each sequence pair and a total of 1492 positions were taken in the final dataset for construction of phylogenetic trees. Phylogenetic reconstruction and validation were performed using neighbour-joining (NJ) method [32] (Fig 1) based on bootstrap analysis with 1000 replications using Jukes-Cantor [33] distance model. Both maximum-likelihood (ML) [34] and minimum-evolution (ME) [35] methods were employed to test the robustness of the trees (Figure A in S1 File). Multi locus sequence analysis (MLSA) was performed using single copy genes which include *gyr*B (1200 bp), *dna*J (1000 bp), *atp*G (400 bp), and *rpo*B (1200 bp). PCR primers and conditions are given (Table A in S1 File). All PCR products were gel purified, cloned into pTZ57R/T vector and sequenced using vector specific primer set (M13F/M13R). Sequences obtained were searched for similarity level using BLASTN, concatenated, and phylogeny was inferred by constructing NJ tree with 1000 bootstrap resampling (Figure B in S1 File).

### Genotypic characterization

Molar G+C content was (mol %) determined following the thermal denaturation method [36]. DNA-DNA hybridization was carried out between strain KAs 5-3^T^ and reference type members (*P*. *mexicana* AMX 26B^T^, *P*. *japonensis* 12-3^T^, *P*. *indica* P15^T^, *P*. *suwonensis* 4M1^T^, *P*. *wuyuanensis* XC21-2^T^, *P*. *putridarboris* WD12^T^, and *P*. *daejeonensis* TR6-08^T^) using a thermal denaturation procedure involving SYBR green dye-DNA binding method [37]. Optimum renaturation temperature (*T*_*OR*_) was calculated and hybridization was performed as described by Mohapatra *et al*. [38]. DNA-DNA hybridization value < 70% or difference in T_m_ values of 5°C or higher was considered as the cut-off for distinct microbial species [39].

### Phenotypic and chemotaxonomic characterization

Morphological, physiological, biochemical, and chemotaxonomic characterization of the strain KAs 5-3^T^ and reference type strains (*P*. *mexicana* AMX 26B^T^, *P*. *japonensis* 12-3^T^, *P*. *indica* P15^T^, *P*. *daejeonensis* TR6-08^T^, *P*. *suwonensis* 4M1^T^, *P*. *putridarboris* WD12^T^, and *P*. *wuyuanensis* XC21-2^T^) were performed by routine cultivation on LB or MSM as appropriate at 30°C. Cell morphology was examined under bright-field (1000 X oil immersion, Olympus) and scanning electron microscopes (SEM-1400; JEOL). For SEM study, cells were fixed with 0.2% (v/v) glutaraldehyde (EM grade, Sigma) in 0.1 mM phosphate buffer saline (PBS), serially dehydrated with ethanol (30 to 100%) (v/v), placed on poly-L-lysine coated cover glass, and viewed under SEM after gold coating (Figure C in S1 File). Motility was tested by flagella staining protocol of Kodaka *et al*. [40]. Temperature sensitivity was assessed at 10–42°C (with increments of 5°C from 10–25°C and 2°C from 26–42°C). Sensitivity towards various pH (3.0–10.0, with increments of 1.0 pH unit) was investigated using appropriate buffer system [pH 3–5 (0.1 M citric acid/0.1 M sodium citrate), pH 6–8 (0.1 M KH_2_PO_4_/0.1 M NaOH, pH 9–10 (0.1 M NaHCO_3_/0.1 M Na_2_CO_3_)] in LB broth, where no significant pH change of the medium was noticed after autoclaving. NaCl tolerance [0–10% (w/v) with increments of 0.5%] was examined in LB broth, where appropriate volume of NaCl was added (from 0–5%) to the autoclaved medium from a sterile stock solution (20%, w/v). For > 5% of NaCl concentrations, the culture medium was prepared in the double strength (2 X) to avoid the dilution done with the addition of higher NaCl stock solution. For sensitivity towards temperature, pH, and NaCl concentrations, cellular growth was assessed by measuring absorbance (growth optical density, OD 600 nm) at 0, 12, 24, and 48 h. Tests for catalase, oxidase, nitrate reduction to N_2_, utilization of gelatin, esculin, citrate, and urea were performed following the standard procedures [41–43]. Other biochemical properties were studied using API 20NE kit (Bio-Merieux) at 30°C for 24–48 h and GEN-III microplate (Biolog) following the manufacturer’s instructions and are presented in Table 1. Gram-staining was performed using Gram staining kit (HiMedia). Susceptibility towards various antibiotics was tested following disc diffusion susceptibility method [44] involving commercially prepared paper antibiotic disks (HiMedia, India): cefixime (5 μg), ceftriaxone (30 μg), amikacin (30 μg), cefotaxime (30 μg), chloramphenicol (30 μg), ofloxacin (5 μg), polymyxin-B (300 units), tetracycline (30 μg), ciprofloxacin (5 μg), and erythromycin (15 μg). Freshly grown bacterial cultures (approximately 2×10^7^ CFU/mL) were spreaded onto the surface of Mueller-Hinton (MH) agar plates and are incubated for 18–24 h at 30°C. The zones of growth inhibition around each antibiotic disks were correlated to the susceptibility of the isolate using the criteria published by the clinical and laboratory standards institute (CLSI, formerly the National Committee for Clinical Laboratory Standards or NCCLS) [45]. Minimum inhibitory concentration (MIC) of As and various heavy metals was evaluated by growing the cells in LB supplemented agar medium under aerobic condition by following the plate dilution protocol of Zhu *et al*. [46]. Increasing concentrations of As [0.1–200 mM] (As^3+^ as NaAsO_2_ and As^5+^ as Na_2_HAsO_4_) or heavy metals [0.1 to 30 mM] (Cd^2+^as CdCl_2_, Co^2+^ as CoCl_2_, Cu^2+^ as CuSO_4_, Fe^3+^as FeCl_3_, Hg^2+^ as HgCl_2_, Cr^6+^ as K_2_Cr_2_O_7_, Se^6+^ as Na_2_SeO_4_, Ni^2+^ as NiCl_2_, Zn^2+^as ZnCl_2_) were amended into the medium and medium without any heavy metal was treated as control. The lowest concentration of metals, which inhibited cellular growth completely, was considered for MIC evaluation (Table 2). Strains of *Escherichia coli* NCIM 2931^T^ and *Cupriavidus metallidurans* DSM 2839^T^ were used as negative and positive control respectively, as the strains are found to have the lowest and highest resistance respectively to the heavy metals tested.

**Table 1:** Phenotypic characteristics that differentiate strain KAs 5-3^T^ from phylogenetically related type strains of *Pseudoxanthomonas* species. **Strains**: 1, KAs 5-3^T^; 2, *P*. *mexicana* AMX 26B^T^; 3, *P*. *japonensis* 12-3^T^; 4, *P*. *indica* P15^T^; 5, *P*. *daejeonensis* TR6-08^T^; 6, *P*. *suwonensis* 4M1^T^; 7, *P*. *wuyuanensis* XC21-1^T^, 8, *P*. *putridarboris* WD12^T^. +; Positive, -; Negative, W; Weak, and ND; No data available. GW; groundwater, HCHD; hexachlorocyclohexane dumpsite, SAS; saline-alkali soil, RT; rotten tree.

Characteristic	1	2	3	4	5	6	7	8
Habitat	GW	Sludge	Soil	HCHD	Soil	Compost	SAS	RT
Motility	-	+	+	+	+	-	+	+
Catalase	+	+	-	+	+	+	+	+
Oxidase	+	+	+	+	+	-	+	+
**Growth**								
Opt. (^0^C)	30	28	28	28	30	30	35	37
10 ^0^C	+	+	+	-	-	+	+	-
40 ^0^C	-	-	-	-	-	+	-	+
pH	6–8	6–9	6–9.5	6–8	7–9	ND	6–7	6–8
NaCl (%)	0.5–5	0.5–4	0.5–3	1–4	1–5	ND	0.5–5	0–3
Nitrate to N_2_	+	-	-	-	-	-	-	-
**Assimilation**								
Esculin	+	+	+	+	-	+	+	+
Casein	+	+	+	-	+	-	+	+
Gelatin	+	+	+	-	W	+	+	+
Urea	+	-	-	-	-	-	-	-
Tween 80	-	+	+	+	-	-	+	+
Arabinose	-	-	-	-	+	+	+	+
Mannose	-	+	-	+	-	-	-	-
NAG	+	-	+	-	+	+	+	+
Maltose	-	+	+	+	+	+	-	+
Gluconate	-	+	-	-	-	+	-	-
Caprate	-	-	-	-	-	-	-	-
Adipate	+	-	-	-	+	-	-	-
Malate	+	+	+	+	-	+	+	-
Citrate	+	+	+	-	+	-	-	-
β-galactosidase	+	+	+	-	+	+	+	-
β-glucosidase	+	-	-	-	+	-	-	+
G+C (mol %)^*^	64.9	67.8±2	65.2±1	62.9±2	68.7±0.4	67.6±1	66.2	69.1

*G+C (mol %) data taken from Thierry *et al*., [3], Kumari *et al*., [13], Yang *et al*., [7], Weon et al., [8], Li et al., [14], and Lee et al., [17] respectively.

**Table 2:** Minimum inhibitory concentration (MIC) of As and other heavy metals tested for strain KAs 5-3^T^ and reference type strains. **Strains:** 1, KAs 5-3^T^; 2, *P*. *mexicana* AMX 26B^T^; 3, *P*. *japonensis* 12-3^T^; 4, *P*. *indica* P15^T^; 5, *P*. *daejeonensis* TR6-08^T^; 6, *P*. *suwonensis* 4M1^T^; 7, *P*. *wuyuanensis* XC21-1^T^; 8, *P*. *putridarboris* WD12^T^; 9, *E*. *coli* NCIM 2931^T^; 10, *C*. *metallidurans* DSM 2839^T^.

Heavy metals [mM]	Bacterial strains									
	1	2	3	4	5	6	7	8	9	10
**Co**^**2+**^	5.0	2.5	3	2.5	2.5	3.0	2.5	2.0	2.0	3.0
**Ni**^**2+**^	3.0	2.5	2.5	2.5	2.5	3.0	2.5	2.0	2.0	2.5
**Cr**^**6+**^	3.0	3.0	2.5	2.5	2.5	2.0	2.0	2.0	1.5	4.0
**Cu**^**2+**^	5.0	2.5	2.5	2.0	2.0	2.0	2.0	5.0	2.5	3.5
**Se**^**6+**^	10.0	2.5	3.0	2.0	2.0	2.0	2.5	3.0	4.0	15.0
**Hg**^**2+**^	2.0	1.0	1.5	1.0	1.0	1.0	1.0	1.0	0.5	2.0
**Zn**^**2+**^	3.5	2.5	2.5	2.5	2.5	2.5	2.5	2.5	2.5	3.0
**Cd**^**2+**^	3.0	2.0	2.0	2.0	2.0	2.0	2.0	2.0	2.5	2.5
**As**^**5+**^	150.0	1.5	2.0	1.5	1.5	2.0	1.0	1.0	3.0	10.0
**As**^**3+**^	20.0	1.0	1.5	1.0	0.5	1.0	1.0	0.0	1.0	4.5
**Fe**^**3+**^	20.0	5.0	5.0	5.0	5.0	5.0	2.5	5.0	10.0	20.0

The analysis of cellular fatty acid methyl esters (FAMEs) was performed after growth of bacterial strains (KAs 5-3^T^, *P*. *mexicana* AMX 26B^T^, *P*. *japonensis* 12-3^T^, *P*. *indica* P15^T^, *P*. *suwonensis* 4M1^T^, *P*. *wuyuanensis* XC21-2^T^, *P*. *putridarboris* WD12^T^, and *P*. *daejeonensis* TR6-08^T^) on Tryptic Soy agar (TSA) for 24 h at 30°C. One loopful of bacterial colony was harvested at exponential phase, subjected to saponification, methylation, and extraction. Fatty acids were determined by Microbial ID using the fully automated GC Sherlock Microbial Identification System (MIDI) using MIDI standard procedures [47]. Isoprenoid quinones were extracted from overnight grown culture following the procedure of Komagata & Suzuki [48] and analysed using high performance liquid chromatography (HPLC, Agilent 1100; column: Sorbax C18 reverse phase, Agilent), where methanol: isopropanol (2:1, v/v), was used as mobile phase with peak detection at 275 nm. The ubiquinone fractions were separated and identified by liquid chromatography-mass spectrometry (LC-MS, WATERS 2695) in a positive-mode electrospray analysis. Polar lipids were extracted and analyzed by two-dimensional TLC following protocol of Komagata & Suzuki [48] (Figure D in S1 File). Polyamines were extracted as described by Kumari *et al*., [13] and analysed by TLC (Silica gel 60 F254, 20×20 cm, Merck, Germany).

### Utilization of carbon substrates, electron acceptors, and As-reductive growth

**Fig 2:**
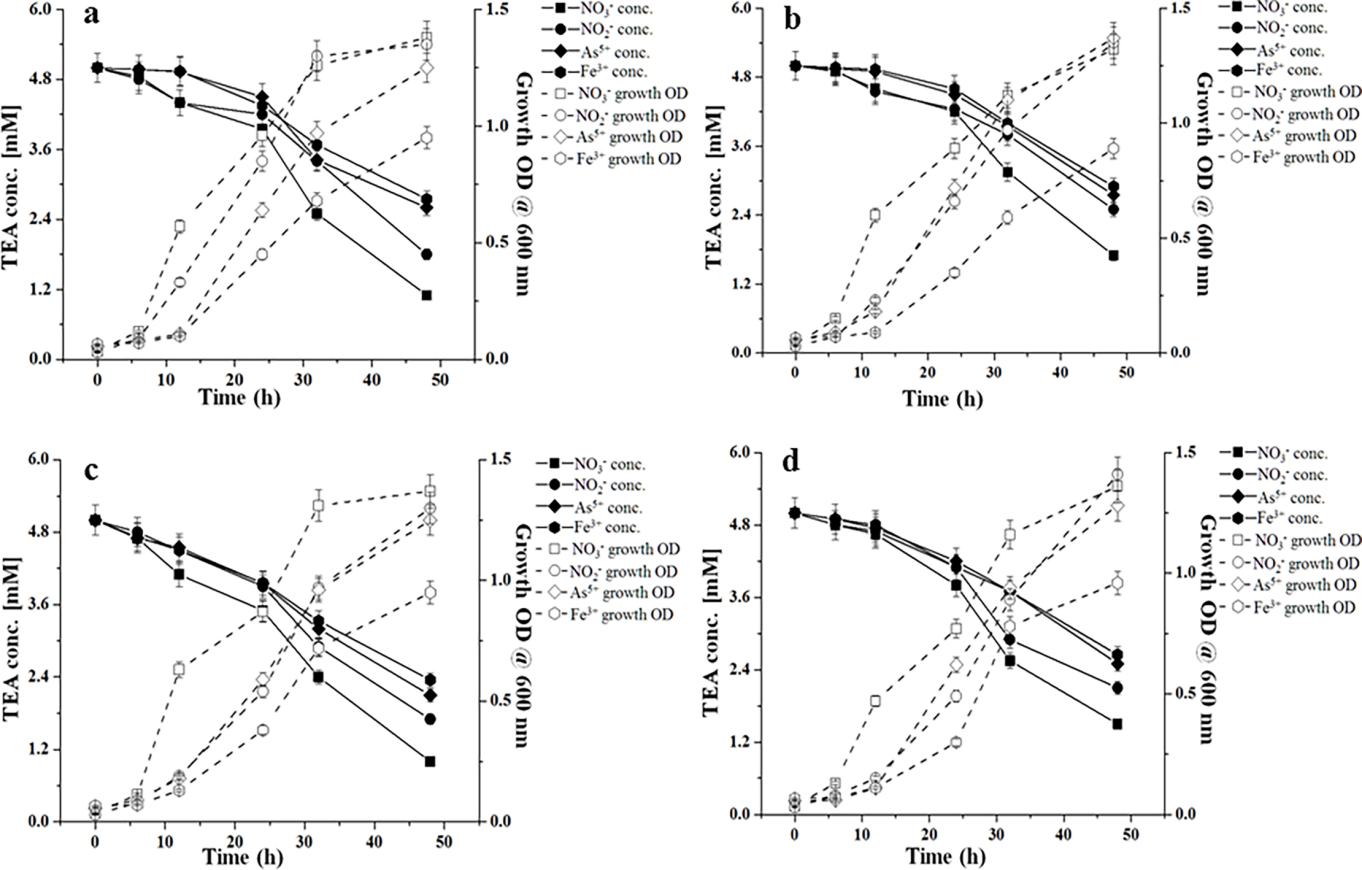
**Growth and reductive use of different electron acceptors (NO**_**3**_^**-**^**, NO**_**2**_^**-**^**, As**^**5+**^**, Fe**^**3+**^**) by strain KAs 5-3**^**T**^ **in the presence of various sugar and hydrocarbon sources as principal carbon substrates**: a) glucose, b) lactate, c) dodecane, and d) pentadecane.

To test the utilization of different carbon substrates by strain KAs 5-3^T^, a range of hydrocarbon compounds (benzene, toluene, xylene, catechol, benzoic acid, naphthalene, phenanthrene, anthracene, pyrene, dodecane, pentadecane, hexadecane, nonadecane, docosane) were amended into MSM medium at a concentration of 500 μM. Freshly grown cell suspension (MSM culture medium) was centrifuged at 10,000 rpm for 5 min, washed twice with 0.85% saline, resuspended in the MSM (without any amendment), inoculated (1%, v/v) into the medium (OD_600_ 0.03–0.05 at t_0_), and incubated for 72 h at 30°C. Growth was monitored at regular intervals by measuring colony forming unit (CFU)/mL, by plating 0.1 mL of the culture onto MSM plates supplemented with respective hydrocarbon sources. Utilization of various terminal electron acceptors (TEAs) was tested following anaerobic growth (OD at 600 nm) with As^5+^ (5 mM), Fe^3+^ (5 mM), NO_3_^-^ (5 mM), NO_2_^-^ (5 mM) or SO_4_^2-^ (5 mM) in MSM [37] as alternate electron acceptors following addition of either sugar substrates (glucose or lactate, 20 mM each) and hydrocarbons (pentadecane or naphthalene, 750 μM each) as the sole carbon/energy source (Fig 2). Medium with added TEAs and without any inoculum was used as abiotic control. The concentration of TEAs in growth medium was measured in duplicate at regular intervals using standard procedures [43, 49–51]. Cytosolic As^5+^ reduction was also checked by growing strain KAs 5-3^T^ in MSM supplemented with carbon sources (as described above) and incubated at 30°C for 24 h. The growth parameters and rate of reduction of As^5+^ were calculated by checking growth OD (at 600 nm) and residual As^5+^ concentration in the medium by spectrophotometric method [52] and validated by atomic absorption spectrophotometer (AAS; PinAAcle900H, Perkin Elmer).

### Functional gene-based analysis

**Fig 3:**
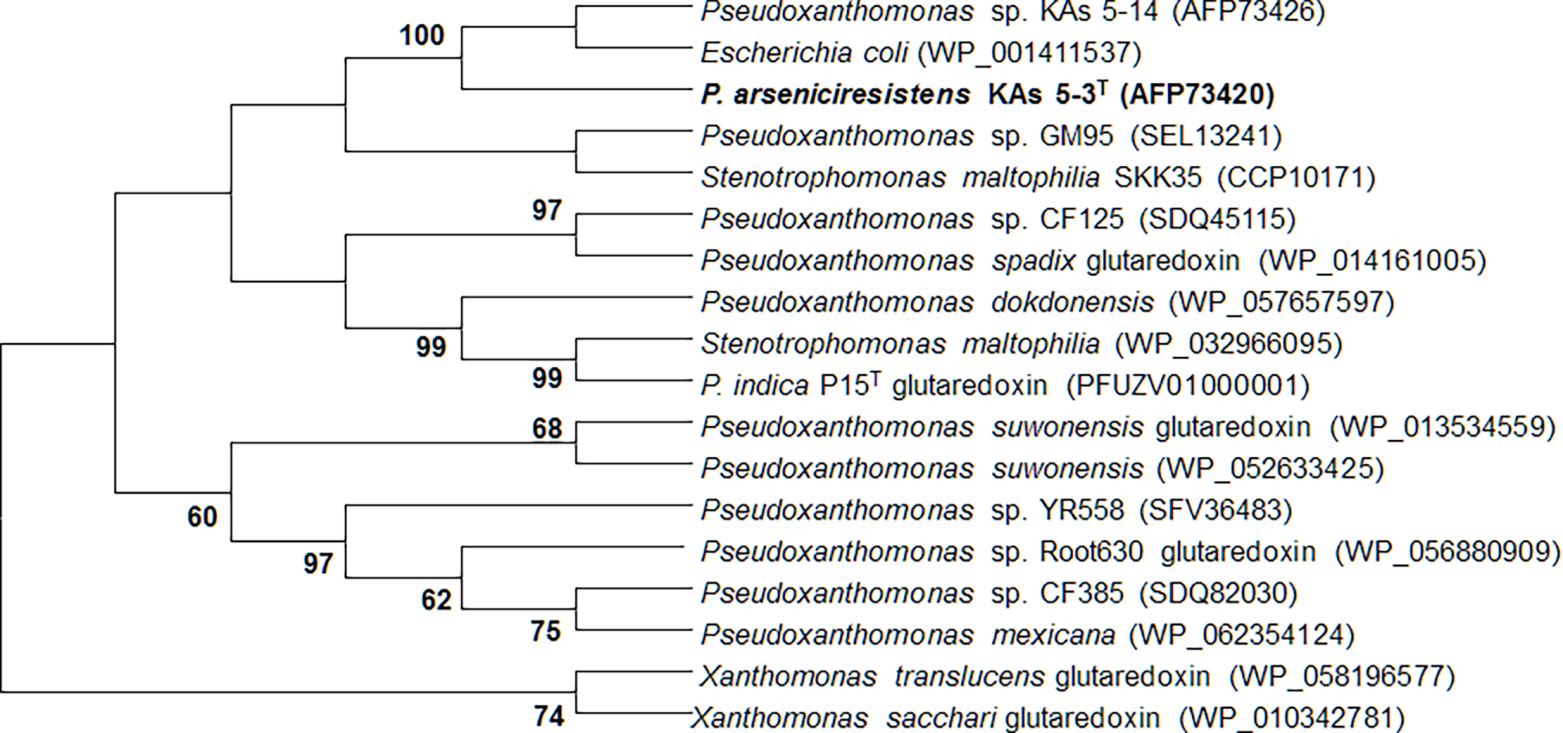
Neighbor-joining phylogenetic tree of genes encoding arsenate reductase (*ars*C) of KAs 5-3^T^ with similar sequences (>90% identity) retrieved from NCBI database. Bootstraps (1000 replications) of above 50% are shown at each branch points. The sequences obtained in this study are highlighted in bold, sequence accession numbers are in parentheses.

**Fig 4:**
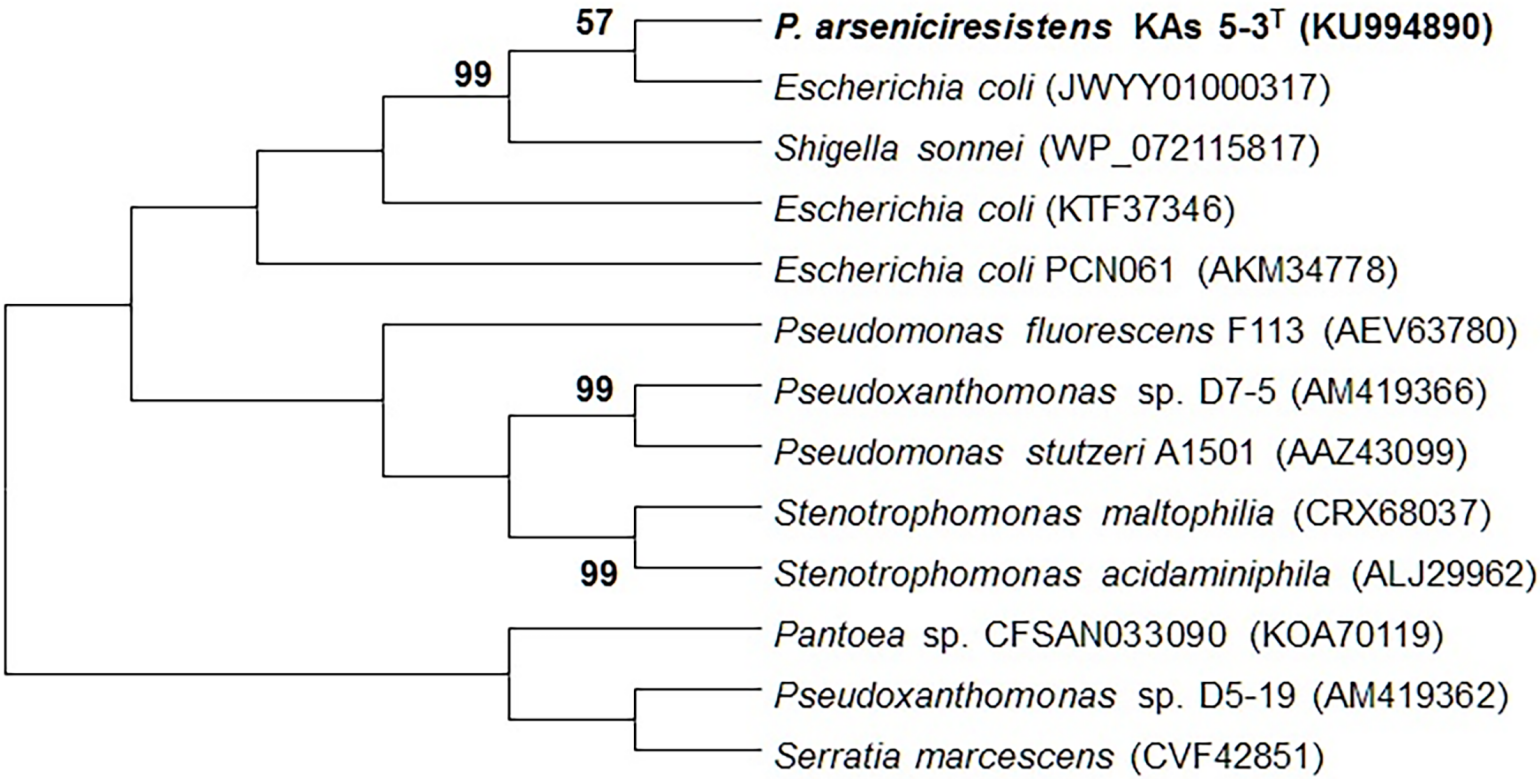
Neighbor-joining phylogenetic tree of genes encoding dissimilatory nitrate reductase (*nar*G) of KAs 5-3^T^ with similar sequences (>90% identity) retrieved from NCBI database. Bootstraps (1000 replications) of above 50% are shown at each branch points. The sequences obtained in this study are highlighted in bold, sequence accession numbers are in parentheses.

Genes responsible for cytosolic As^5+^ reduction (*ars*C), dissimilatory nitrate- (*nar*G) and nitrite reduction (*nir*S) were also amplified through PCR based approach (Table A in S1 File). All PCR products were gel purified, cloned and sequenced (as described above for MLSA). Nucleotide sequences obtained were searched for similarity level using BLASTN. The corresponding nucleotides were translated to amino acids in ExPasy tool [53] using appropriate open reading frames (ORFs) and searched in BLASTP, (nr database) excluding options for uncultured/environmental sequences and including option for type material. Conserved domain was predicted through CDD database and phylogeny was inferred through neighbour-joining method (Figs 3 and 4) considering the translated amino acid sequence of strain KAs 5-3^T^ and similar sequences (>90% similarity value). The nucleotide sequences were analyzed for GC content (mol %), GC % deviation from their respective genomes (Table B in S1 File) as well as p-distance calculations through MEGA 7.0. Phylogenetic network analysis was performed using SplitsTree software [54] (Figures E and F in S1 File).

The GenBank accession numbers for the 16S rRNA, *gyr*B, *atp*G, *dna*J, *rpo*B, *ars*C, *nar*G, and *nir*S gene sequences of strain KAs 5-3^T^ are JX173988, KX827793, KX827799, KX827796, KX880497, JX110552, KU994890 and KY563659 respectively.

## Results and discussion

### 16S rRNA gene phylogeny and multi locus sequence typing

Comparison of nearly complete (1,495 nucleotides) 16S rRNA gene sequence indicated taxonomic affiliation of strain KAs 5-3^T^ to the genus *Pseudoxanthomonas*, with highest sequence similarity to the type strains of *P*. *mexicana* AMX 26B^T^ (99.25%), *P*. *japonensis* 12-3^T^ (98.9%), followed by *P*. *putridarboris* WD-12^T^ (98.02%), *P*. *indica* P15^T^ (97.27%), *P*. *wuyuanensis* XC21-2^T^ (97.12%), *P*. *suwonensis* 4M1^T^ (97.0%), and *P*. *daejeonensis* TR6-08^T^ (96.99%). The NJ phylogenetic analysis showed that strain KAs 5-3^T^ formed a coherent cluster of monophyletic pattern with the type strains of *P*. *mexicana* AMX 26B^T^ and *P*. *japonensis* 12-3^T^ (bootstrap support of 100.0%) and claded to the type members of *Pseudoxanthomonas* (Fig 1). Both ML and ME phylogenetic reconstruction methods indicated a consistent tree topology cladding strain KAs 5-3^T^ to the AMX 26B^T^, 12-3^T^, P15^T^, and WD12^T^ as the nearest phylogenetic neighbours, while the clade comprising the near distant members of the strain KAs 5-3^T^ was only supported by either of the methods (Figure A in S1 File). On the basis of high percentage of 16S rRNA gene sequence homology and coherent monophyletic cladding of strain KAs 5-3^T^, the type strains *P*. *mexicana* AMX 26B^T^,*P*. *japonensis* 12-3^T^, *P*. *indica* P15^T^, and *P*. *putridarboris* WD12^T^ are inferred to be the closest phylogenetic neighbours. Multi locus sequence typing (MLST) involving various hose-keeping genes have been employed as a taxonomic marker for species level comparisons and clonal relationship [55, 56]. Sequence analysis of *gyr*B, *dna*J, *rpo*B, and *atp*G genes of strain KAs 5-3^T^ showed > 92.0% sequence similarity to the type strains *P*. *mexicana* AMX 26B^T^ and *P*. *japonensis*12-3^T^ but formed a separate clade in the NJ phylogenetic reconstruction (Figure B in S1 File) indicating its non-clonal nature and species distinction from both the closest phylogenetic neighbours.

### Genotypic characterization

The genomic G+C content of strain KAs 5-3^T^ was found to be 64.9 mol %, this value is within the range for the genus *Pseudoxanthomonas* [2, 3]. It has been strongly emphasized that inter-species differentiation should be evaluated by using DNA–DNA hybridization (DDH) studies [56]. The levels of DNA-DNA relatedness of the strain KAs 5-3^T^ with *P*. *mexicana* AMX 26B^T^, *P*. *japonensis* 12-3^T^, *P*. *suwonensis* 4M1^T^, *P*. *wuyuanensis* XC21-2^T^, *P*. *indica* P15^T^, *P*. *daejeonensis* TR6-08^T^, *P*. *putridarboris* WD12^T^ were calculated to be 35.7%, 35.5%, 35.5%, 35.0%, 32.5%, 32.0%, and 22.1% respectively. Since DNA-DNA relatedness < 70% is considered to be the cut-off value for species delineation, KAs 5-3^T^ is unambiguously proposed to be a novel species [57].

### Phenotypic and chemotaxonomic characterization

Culture characteristics revealed that on LB agar plates, colonies of strain KAs 5-3^T^ were creamy to pale yellow, circular, with entire margin and a diameter range of 1–2 mm after 24–48 h. Cells were Gram-stain-negative, rod-shaped, aerobic to facultative anaerobic, non-motile, catalase and oxidase positive, with a cell size of 1.2–1.5 μm length × 0.3–0.5 μm width (Figure C in S1 File). The strain was found to grow well at temperature range of 10–38°C (optimum at 28–32°C), pH range of 6.0–8.0 (optimum at 7.0) and over a broad spectrum of NaCl concentrations (0.5–5%; optimum of 1%) and growth did not occur without NaCl in the medium. The other details of phenotypic characteristics of the strain KAs 5-3^T^ are presented in the species description and Table 1. Compared with other type members of the same genus (*Pseudoxanthomonas*), strain KAs 5-3^T^ exhibited phenotypic differences (Table 1). The strain KAs 5-3^T^ showed ability to reduce nitrate to N_2_, assimilate esculin, casein, gelatin, urea, adipate, malate, citrate, and N-acetyl glucosamine (NAG) and showed negative response for tween 80, arabinose, mannose, gluconate, and caprate. The catalase-, oxidase-positive, mesophilic, slightly alkalophilic and heterotrophic growth pattern confirmed relatedness of KAs 5-3^T^ to the same genus [13]. The differential phenotypic properties *viz*., motility, assimilation of tween 80, urea, maltose, adipate, and production β-glucosidase confirmed the species level distinction of KAs 5-3^T^ from the compared *Pseudoxanthomonas* members. In comparison with the phylogenetic neighbours, strain KAs 5-3^T^ showed considerably higher resistance towards several metals Co^2+^, Cu^2+^, Se^6+^, Fe^3+^, As^3+^, and As^5+^ (Table 2). The strain’s ability to withstand Fe^3+^ was comparable to multi-metal resistant *C*. *metallidurans* and for As species, it was highest amongst all the strains tested.

The predominant quinone of the strain KAs 5-3^T^ was found to be Q8. This seems to be a familiar character as prevalence of Q8 was previously reported as the major quinone in members of the genus *Pseudoxanthomonas* [3, 5, 13]. The overall FAME profile of the strain KAs 5-3^T^ was found to be consistent to that of other type strains compared with some observed quantitative differences (Table 3). The major cellular fatty acids (> 5% of the total fatty acids) of strain KAs 5-3^T^ consisted of C_15:0_ (37.4%), C_16:0_ iso (12.6%), C_17:1_ iso ω9c (10.5%), C_15:0_ anteiso (9.5%), C_11:0_ iso 3-OH (8.5%), and C_16:1_ ω7c/ C_16:1_ ω6c (7.5%). The overall FAME profile was similar with the type strains compared, but the differential presence of C_11:0_ anteiso, C_16:0,_ as well as absence of C_15:1_ iso F and C_16:1_ iso H distinguished the strain KAs 5-3^T^ from the reference type strains.

**Table 3:** Cellular fatty acid profiles of strain KAs 5-3^T^ and related type members of the genus *Pseudoxanthomonas*. **Strains:** 1, KAs 5-3^T^; 2, *P*. *mexicana* AMX 26B^T^; 3, *P*. *japonensis* 12-3^T^; 4, *P*. *indica* P15^T^; 5, *P*. *daejeonensis* TR6-08^T^; 6, *P*. *suwonensis* 4M1^T^; 7, *P*. *wuyuanensis* XC21-2^T^; 8, *P*. *putridarboris* WD12^T^.

Fatty acids*	Strains							
Saturated	1	2	3	4	5	6	7	8
C_10:0_	-	-	-	0.5	-	0.2	-	0.9
C_16:0_	1.9	0.7	0.6	2.0	0.3	1.2	0.8	9.0
**Unsaturated**								
C_18:1_ ω9c	0.7	0.5	0.6	0.3	0.9	-	-	0.8
**Methyl branched**								
C_10:0_ iso	0.4	-	-	0.5	-	0.5	-	-
C_11:0_ iso	3.1	5.4	5.1	4.2	4.5	7.2	5.9	4.2
C_11:0_ anteiso	2.8	-	-	0.3	-	0.5	1.1	0.9
C_14:0_ iso	2.4	2.5	3.2	3.2	2.2	2.9	1.4	3.5
C_15:1_ iso F	-	1.3	1.6	1.9	-	1.5	0.7	-
C_15:0_ iso	37.4	39.2	35.6	27.9	40.5	31.7	28.5	14.5
C_15:0_ anteiso	9.5	2.6	3.8	5.6	6.5	12.8	3.6	6.2
C_16:1_ iso H	-	3.9	4.2	0.2	-	0.4	1.1	1.5
C_16:0_ iso	12.6	9.8	12.5	18.3	6.9	10.9	16.8	20.5
C_17:0_ iso	1.5	4.5	3.7	3.1	1.2	2.8	3.6	-
C_17:0_ anteiso	-	0.5	0.7	1.2	0.8	1.2	1.6	1.2
**Hydroxy**								
C_11:0_ iso 3-OH	8.5	6.4	4.9	6.6	4.5	7.2	5.5	4.5
C_12:0_ iso 3-OH	2.2	0.9	0.4	1.1	-	1.3	1.2	1.8
**Summed feature**								
C_16:1_ ω7c/ C_16:1_ ω6c	6.5	5.7	6.1	4.9	6.9	4.5	2.5	7.5
C_18:1_ ω6c	-	1.1	0.79	0.4	1.1	0.8	-	1.0
C_17:1_ iso ω9c	10.5	19.5	19.5	17.6	20.0	11.6	18.1	11.5

*All strains were cultured and grown under the same conditions. The values shown are percentages of total fatty acids.

The polar lipid profile of the strain KAs 5-3^T^ was found to be consisting of diphosphatidylglycerol (DPG), phosphatidyldimethylethanolamine (PDE), phosphatidylcholine (PC), and unknown phospholipids (PL1, PL2, PL3). The presence of DPG, PDE and PL1 was found to be consistent in all the compared members (except *P*. *indica* P15^T^), indicating the affiliation of strain KAs 5-3^T^ to the members of the genus *Pseudoxanthomonas*. The appearance of spot corresponding to PC and absence of PE, unknown lipids (UL1, UL2) uniquely distinguished the strain KAs 5-3^T^ from all the compared members (Figure D in S1 File).

### Utilization of carbon substrates, electron acceptors, and As-reductive growth

Cells of the strain KAs 5-3^T^ were found to utilize catechol, naphthalene, dodecane, and pentadecane as sole carbon sources. Among various tested electron acceptors, strain KAs 5-3^T^ showed growth on As^5+^, NO_3_^-^, NO_2_^-^, and Fe^3+^, while no growth was observed in SO_4_^2-^. But, the preferential pattern [net reduction of each added TEA (mM) vs time] was found to be NO_3_^-^ > NO_2_^-^ > As^5+^ > Fe^3+^. The growth of the strain while growing under these preferred electron acceptors showed that after 48 h, it reduced NO_3_^-^ preferably (5 mM to avg. of 1 mM) followed by NO_2_^-^ (5 mM to avg. of 2.0 mM), As^5+^ (5 mM to avg. of 2.5 mM), and Fe^3+^ (5 mM to avg. of 2.8 mM) (Fig 2). Substantial growth [with a maximum growth OD of 1.2–1.3, μ = 0.11 h^-1^] along with the formation of As^3+^ in the aqueous medium, confirmed its reductive transformation ability. Cells of strain KAs 5-3^T^ were also found to reduce As^5+^ (from 1 mM to 0.2 mM) within 30 h of aerobic growth with the concomitant release of As^3+^ in the supernatant, indicating its potential of cytosolic reduction of As^5+^. The ability of *Pseudoxanthomonas* members to metabolize alkyl and aromatic hydrocarbons (BTEX, chrysene, and phenanthrene) and degrade pollutants has been recently studied [20, 21, 58–60]. The As-rich groundwater of Bengal basin harbours low amount of petroleum-derived hydrocarbons (that naturally seeps into the groundwater from deeper mature sediments), presence and hydrocarbon metabolizing activity of *Pseudoxanthomonas* strains is highly justified [26, 61, 62]. Except for *P*. *kausinghensis* and *P*. *dokdonensis*, *Pseudoxanthomonas* type members have been known to reduce nitrite. Thus, the ability of strain KAs 5-3^T^ to preferentially utilize NO_3_^-^ over NO_2_^-^ is considered to be a unique metabolic character, distinguishing the strain from its closest relatives. Strain’s ability in utilizing diverse electron acceptor sources, thus corroborates its potential to dwell at the interface of aerobic-anaerobic zones of groundwater [26, 62–65].

### Functional gene-based analysis

The presence of cytosolic As^5+^ reductase (*ars*C; 118 AA), nitrate reductase (*nar*G; 214 AA) and nitrite reductase (*nir*S; 146 AA) were noted for the strains KAs 5-3^T^ but not for the other closest related strains. BLASTP search showed highest identity (100%) of *ars*C and *nar*G genes to the same genes from *Escherichia coli* followed by several *Pseudoxanthomonas* and other *Xanthomonas*, while the sequence of *nir*S showed highest similarity with *Pseudoxanthomonas helianthi* roo 10. Elaborate phylogenetic analysis was conducted for the *ars*C and *nar*G genes. Phylogenetic analysis (Figs 3 and 4), p-distance matrix based net amino acid substitution (Figures E and F in S1 File), and phylogenetic neighbour network (Figures E and F in S1 File) showed a close phylogenetic proximity among KAs 5-3^T^ and *E*. *coli* with respect to both of these genes. The data further indicated presence of similar mutational (insertion/deletion) events in these genes from the organisms, thus suggesting their possible transfer through horizontal gene transfer events. So, the observed phylogenetic incongruence between these functional genes and 16S rRNA gene was further studied with respect to GC mol %. Measure of unrelated GC mol % of the functional genes in the genome of organisms is considered to be the possible site of gene transfer events [24, 66, 67]. Hence, GC content (mol % and mol % deviation) of both the genes was compared with the genomic GC mol % for *Pseudoxanthomonas* reference genomes (Table B in S1 File). The GC mol % of both *ars*C and *nar*G of strain KAs 5-3^T^ were close to the genomic GC content of *E*.*coli* genomes, but not to the genomes of any of the nearest *Pseudoxanthomonas* members, further supporting the possibility of horizontal gene transfer events [68, 69]. Unlike, nitrite reduction, a universal property for the genus *Pseudoxanthomonas*; nitrate reduction by strain KAs 5-3^T^, is a unique trait.

The abilities to utilize multiple hydrocarbons, different electron acceptors with As^5+^ reduction abilities and genetic validation of this potential clearly demonstrated the metabolic flexibility of the strain. Alluvial aquifer of West Bengal is oligotrophic in nature with low dissolved carbon, low oxygen tension, fluctuating availability of electron donors and acceptors, with a low concentration of naturally derived hydrocarbons [26, 38, 61, 62]. Considering the overall hydrogeochemistry of West Bengal groundwater, the metabolic versatility of the strain KAs 5-3^T^ seems highly justified for its competitive niche adaptation.

### Emended description of the genus *Pseudoxanthomonas* Finkmann et al. 2000 emend. Lee et al. 2008

As per the descriptions of *Pseudoxanthomonas* by Finkmann et al., emended by Lee et al. (2008) and properties tested in this study, an emended description of the genus *Pseudoxanthomonas* is provided. Type strains of all *Pseudoxanthomonas* species except *P*. *kaohsiungensis*, *P*. *dokdonensis*, and *P*. *arseniciresistens* have no nitrate reduction (to N_2_) ability.

### Description of *Pseudoxanthomonas arseniciresistens* sp. nov.

*Pseudoxanthomonas arseniciresistens* (L. n. *arsenicum*, arsenic; L. part. adj. *resistens*, resisting; N.L. part. adj. *arseniciresistens*, arsenic resisting, referring to the high arsenic resistance of the type strain).

Colonies are creamy to yellow, smooth and circular (1–2 mm on LB agar after 24–48 h at 30 ^o^C). Cells are Gram-stain-negative, and facultative anaerobic rods (~1.5 × 0.5 mm). It grows well at 28–32 ^o^C, pH 6–8 and NaCl concentrations of 0.5–5% (optimum of 1%). Cells are catalase- and oxidase-positive, highly As-resistant and able to reduce arsenate, nitrate as well as nitrite. Cells are positive for hydrolyses of ONPG (beta-galactosidase), beta-glucosidase, esculin, gelatin, casein, utilization of adipate, malate, citrate, N-acetyl glucosamine (NAG), and urea but negative for tween 80, arabinose, mannose, mannitol, maltose, gluconate, and caprate. Among various sugars, it assimilates α-D glucose, D-turanose, D-raffinose, D-sorbitol, D-galactose, sucrose, myo-inositol, and dextrin but does not assimilate α-D lactose, D-maltose, D-trehalose, D-cellobiose, D-fucose, D-mannose, D-salicin, gentiobiose, inosine, tween 40, and 3-methyl glucose. Among sugar acids, it waspositive for the assimilation of α-keto glutaric acid, D-gluconic acid, D-glucuronic acid, D-galacturonic acid, D-lactic acid, D-aspartic acid, D-malic acid, L-malic acid, L-aspartic acid, L-glutamic acid, acetic acid, citric acid, mucic acid, propionic acid, fusidic acid, sodium lactate, amino butyric acid, β-hydroxy butyric acid but negative for α-hydroxy butyric acid, α-keto butyric acid, L-galactonic acid, aceto acetic acid, phenyl acetic acid, and N-acetyl neuraminic acid. Among N-containing compounds, it uses L-glycyl proline, L-alanine, L-serine, D-serine, but unable to use D-glycyl proline, L-arginine, and L-histidine. On Biolog plates, cells of the strain KAs 5-3^T^ shows ability to use glucuronamide, guanidine-HCl, tetrazolium violet, tetrazolium blue, lithium chloride, and potassium tellurite and inability to use sodium bromate. The cells are resistant to erythromycin, but, susceptible to ceftriaxone, cefixime, amikacin, cefotaxime, chloramphenicol, ofloxacin, polymyxin-B, tetracycline, ciprofloxacin, troleandomycin, rifamycin SV, minocycline, lincomycin, vancomycin, nalidixic acid and aztreonam. Cells are able to use hydrocarbons and reduce arsenate through cytosolic reduction. The major cellular fatty acids are C_15:0_, C_16:0_ iso, C_17:1_ iso ω9c, C_15:0_ anteiso, C_11:0_ iso 3-OH and C_16:1_ ω7c/ C_16:1_ ω6c and Q8 as the major isoprenoid quinone. Polar lipids include diphosphatidylglycerol, phosphatidyldimethylethanolamine, phosphatidylcholine, and three unknown phospholipids. Spermidine is the predominant polyamine. The molar G+C content is 64.9 mol %.The type strain, KAs 5-3^T^ (= LMG 29169^T^ = MTCC 12116^T^ = MCC 3121^T^), was isolated from highly As-rich groundwater of Kolsur village, North 24 Pargana of West Bengal, India.

## Conclusion

The phylogenetic, chemotaxonomic and phenotypic analysis supported the affiliation of strain KAs 5-3^T^ to the genus *Pseudoxanthomonas*. The strain KAs 5-3^T^ showed distinguishing physiological, phenotypic as well as molecular characteristic. Multi locus sequence analysis involving four house-keeping genes and DNA–DNA relatedness unambiguously demarcated the species novelty. Dissimilatory reduction of nitrate and nitrite as well as ability to metabolize hydrocarbons and reduce As^5+^ through cytosolic processes highlighted the unique properties of the strain KAs 5-3^T^, which are of ecological significance. On the basis of phenotypic and physiological characteristics, chemotaxonomic analysis, multi locus sequence analysis, and DNA–DNA relatedness data, the isolate represents a novel species of the genus *Pseudoxanthomonas*, therefore, the name *Pseudoxanthomonas arseniciresistens* sp. nov. is proposed.

## Supporting information

S1 File **Table A**, **Details of PCR primers used for 16S rRNA, MLSA, and functional gene analysis**. **Table B**, **GC mol % and dGC mol % (deviation) from their respective genomic GC content of *ars*C and *nar*G sequences (phylogenetically closest) as a measure of horizontal gene transfer event**. **Figure A**, **Phylogenetic tree involving 16S rRNA gene sequences of strain KAs 5-3**^**T**^ **and type members of *Pseudoxanthomonas* species obtained through (a) maximum likelihood (b) and minimum evolution methods**. Bootstraps (1000 resampling) of above 60% are shown at each branch. Genbank accession numbers are presented in parentheses. Bar 0.005 indicates 0.5% substitution. **Figure B**, **Neighbor-joining phylogenetic tree based on Multi Locus Sequence Alignment (MLSA) of four concatenated housekeeping genes: *gyr*B (1200 bp), *dna*J (1000 bp), *atp*G (400 bp), and *rpo*B (1200 bp) of KAs 5-3**^**T**^ **with the *Pseudoxanthomonas* type members**. The percentage of replicate trees in which the associated taxa clustered together in the bootstrap test (1000 replicates) is shown next to the branches. The evolutionary distances were computed and are in the units of the number of base substitutions per site. All ambiguous positions were removed and all codon positions were included for construction of the tree in the final dataset through MEGA 7.0. GenBank accession numbers for the genes of strain KAs 5-3^T^ are: KX827793 (*gyr*B), KX827796 (*dna*J), KX827799 (*atp*G), and KX880497 (*rpo*B). **Figure C**, **Scanning electron micrograph of cells of the strain KAs 5-3**^**T**^**after growth on LB agar plate for 18 h at 30°C**. **Figure D**, **Polar lipid profile of the strain KAs 5-3**^**T**^ **and reference type strain members of *Pseudoxanthomonas* as shown on TLC plate, developed after spraying with 5% ethanolic molybdophosphoric acid lipid detection solvents**; a) KAs 5-3^T^, b) *P*. *mexicana* AMX 26B^T^, c) *P*. *japonensis* 12-3^T^, d) *P*. *daejeonensis* TR6-08^T^, e) *P*. *indica* P15^T^, f) *P*. *suwonensis* 4M1^T^, g) *P*. *wuyuanensis* XC21-2^T^, h) *P*. *putridarboris* WD12^T^. **Figure E**, **Analysis of gene encoding arsenate (As**^**5+**^**) reductase (*ars*C) a) distance matrix for aligned sequence of KAs 5-3**^**T**^ **with related sequences, b) NeighborNet phylogenetic network of *ars*C gene of KAs 5-3**^**T**^ **with related sequences obtained through SplitsTree software**. Colour codes indicate the p-distance value against the specified sequences. Intensity in each branch indicates the similar evolutionary events. Bar, 0.1 indicates extent of evolution (10%) at amino acid level. **Figure F**, **Analysis of gene encoding nitrate (NO**_**3**_^**-**^**) reductase (*nar*G) a) distance matrix for aligned sequence of KAs 5-3**^**T**^ **with related sequences, b) NeighborNet phylogenetic network of nitrate reductase (*nar*G) of KAs 5-3**^**T**^ **with related sequences obtained through SplitsTree software**. Colour codes indicate the p-distance value against the specified sequences. Intensity in each branch indicates the similar evolutionary events. Bar, 0.1 indicates extent of evolution (10%) at amino acid level.
(PDF)

S1 Certificate **A**, Microbial culture collection (MCC) deposition certificate of strain KAs 5-3^T^. **B**, Microbial type culture collection (MTCC) deposition certificate of strain KAs 5-3^T^. **C**, Belgian coordinated culture collection (BCCM) deposition certificate of strain KAs 5-3^T^.
(PDF)
