# Supplementary material for: Taxonomy and physiology of Pseudoxanthomonas arseniciresistens sp. nov., an arsenate and nitrate-reducing novel gammaproteobacterium from arsenic contaminated groundwater, India
Source: PLoS One. 2018 Mar 20;13(3):e0193718. doi: 10.1371/journal.pone.0193718 (PMC5860741; doi:10.1371/journal.pone.0193718)
Supplement: S1 File — Table A, Details of PCR primers used for 16S rRNA, MLSA, and functional gene analysis. Table B, GC mol % and dGC mol % (deviation) from their respective genomic GC content of arsC and narG sequences (phylogenetically closest) as a measure of horizontal gene transfer event. Figure A, Phylogenetic tree involving 16S rRNA gene sequences of strain KAs 5-3T and type members of Pseudoxanthomonas species obtained through (a) maximum likelihood (b) and minimum evolution methods. Bootstraps (1000 resampling) of above 60% are shown at each branch. Genbank accession numbers are presented in parentheses. Bar 0.005 indicates 0.5% substitution. Figure B, Neighbor-joining phylogenetic tree based on Multi Locus Sequence Alignment (MLSA) of four concatenated housekeeping genes: gyrB (1200 bp), dnaJ (1000 bp), atpG (400 bp), and rpoB (1200 bp) of KAs 5-3T with the Pseudoxanthomonas type members. The percentage of replicate trees in which the associated taxa clustered together in the bootstrap test (1000 replicates) is shown next to the branches. The evolutionary distances were computed and are in the units of the number of base substitutions per site. All ambiguous positions were removed and all codon positions were included for construction of the tree in the final dataset through MEGA 7.0. GenBank accession numbers for the genes of strain KAs 5-3T are: KX827793 (gyrB), KX827796 (dnaJ), KX827799 (atpG), and KX880497 (rpoB). Figure C, Scanning electron micrograph of cells of the strain KAs 5-3Tafter growth on LB agar plate for 18 h at 30°C. Figure D, Polar lipid profile of the strain KAs 5-3T and reference type strain members of Pseudoxanthomonas as shown on TLC plate, developed after spraying with 5% ethanolic molybdophosphoric acid lipid detection solvents; a) KAs 5-3T, b) P. mexicana AMX 26BT, c) P. japonensis 12-3T, d) P. daejeonensis TR6-08T, e) P. indica P15T, f) P. suwonensis 4M1T, g) P. wuyuanensis XC21-2T, h) P. putridarboris WD12T. Figure E, Analysis of gene encoding arsena [file pone.0193718.s001.pdf]

Table A. Details of PCR primers used for 16S rRNA, MLSA, and functional gene analysis

Gene	Primers	Primer Sequence (5´-3´)	Annealing temperature (°C)	Amplicon size (bp)	References
16S rRNA	27F<br>1492R	5’-AGAGTTTGATCCTGGCTCAG-3’<br>5’- GGTTACCTTGTTACGACTT-3’	55.0	1500	[70]
*gyr*B	Up1F<br>Up2R	5’- GAA GTC ATC ATG ACC GTT CTG CAY GCN GGN GGN AAR TTY GA -3’<br>5’- AGC AGG GTA CGG ATG TGC GAG CCR TCN ACR TCN GCR TCN GTC AT-3'	65.0	1260	[71]
*atp*G	atpGF<br>atpGR	5'-TCAAGGAGCGTCTGTCGAT-3'<br>5'-TCTGCCTCGTTGACCTTGA-3'	60.0	405	[72]
*rpo*B	rpoB2F<br>rpoB3R	5-TCAAGGAGCGTCTGTCGA -3'<br>5'-TCTGCCTCGTTGACCTTGA-3'	55.0	1212	[73]
*dna*J	dnaK-F<br>dnaJ-R	5'-CAGATCGAGGTSACCTTCGAC-3'<br>5'-CGTCRYCATMGAGATCGGCAC-3'	54.0	1014	[72]
*ars*C	amlt-42 F<br>amlt-376 R	5-TCGCGTAATACGCTGGAGAT-3'<br>5-ACTTTCTCGCCGTCTTCCTT-3'	53.0	400	[74]
*nar*G	1960 F<br>2659 R	5′ TAY GTS GGC CAR GAR AA-3′<br>5′ TTY TCR TAC CAB GTB GC-3′	58.0	650	[75]
*nir*S	nirSF<br>nirSR	5′ GCN TGY TGG WSN TGY AA-3′<br>5′ TWN GGC ATR TGR CAR TC-3′	58.0	500	[75]

71. Islam E, Sar P Culture-dependent and -independent molecular analysis of bacterial community within uranium ore. J Basic Microbiol. 2011; 4: 1-13.

72. Yamamoto S, Harayama S. PCR amplification and direct sequencing of gyrB genes with universal primers and their application to the detection and taxonomic analysis of *Pseudomonas putida* strains. Appl Environ Microbiol. 1995; 61: 1104-1109.

73. Alexandre A, Laranjo M, Young JPW, Oliveira S. dnaJ is a useful phylogenetic marker for alphaproteobacteria. Int J Syst Evol Microbiol. 2008; 58: 2839-2849.
74. Ferreira-Tonin M, Rodrigues-Neto J, Harakava R, Destéfano SAL. Phylogenetic analysis of *Xanthomonas* based on partial rpoB gene sequences and species differentiation by PCR-RFLP. Int J Syst Evol Microbiol. 2012; 62: 1419-1424.
75. Sun Y, Polischuk EA, Radoja U, Cullen WR. Identification and quantification of arsC genes in environmental samples by using real-time PCR. J Microbiol Methods. 2004; 58: 336-349.

Table B. GC % and dGC % (deviation) from their respective genomic GC content of *ars*C and *nar*G sequences (phylogenetically closest) as a measure of horizontal gene transfer event

**arsenate reductase (*ars*C)**	**GC%**	**dGC% (genomic mean)**
KAs5-3_(AFP73420)	54.46	10.5-15.5 (-)
Pseudoxanthomonas sp. KAs5-14 (AFP73426)	54.70	10.3-15.3 (-)
Pseudoxanthomonas sp. CF125_(SDQ45115)	63.22	1.8-5.8 (-)
P. suwonensis glut (WP_013534559)	66.19	(+)1.2-3.8(-)
P. spadix glut (WP_014161005)	64.86	0.2-5.2 (-)
Pseudoxanthomonas sp. CF385 (SDQ82030)	63.07	2.0-7.0 (-)
Pseudoxanthomonas sp. Root630 glut (WP_056880909)	63.78	1.2-6.2 (-)
P. mexicana (WP_062354124)	62.60	2.5-7.5 (-)
Pseudoxanthomonas sp. YR558_(SFV36483)	61.39	4.6-9.6 (-)
P. dokdonensis (WP_057657597)	61.86	4.2-9.2(-)
Pseudoxanthomonas sp._GM95_(SEL13241)	60.71	4.3-9.3 (-)
P. suwonensis (WP_052633425)	67.24	(+)2.4-2.8(-)
S. maltophilia (WP_032966095)	60.96	4.1-9.1(-)
S. maltophilia SKK35 (CCP10171)	60.86	4.1-9.1(-)
X. translucens glut (WP_058196577)	65.01	(+)0-5.0 (-)
X. sacchari (WP_010342781)	65.01	(+)0-5.0 (-)
S. maltophilia Ab55555 (EJP78927)	60.53	4.5-9.5(-)
E. coli (WP_001411537)	54.34	(+) 14.3-5.6(-)
**dissimilatory nitrate reductase (*nar*G)**	**GC%**	**dGC% (genomic mean)**
KAs_5-3_(KU994890)	59.87	5.2-7.2 (-)
*Pseudoxanthomonas*_sp._D7-5_(AM419366)	56.15	8.9-13.9 (-)
*Pseudoxanthomonas*_sp._D5-19_(AM419362)	59.27	5.8-10.8 (-)
*Escherichia_coli*_PCN061_(AKM34778)	56.64	8.4-13.4 (-)
*Escherichia_coli*_(JWYY01000317)	58.51	6.5-11.5 (-)
*Shigella_sonnei*_(WP_072115817)	57.55	7.5-12.5 (-)
*Pseudomonas_fluorescens*_F113_(AEV63780)	57.12	7.8-12.8 (-)
*Pseudomonas_stutzeri*_A1501_(AAZ43099)	56.62	8.4-13.4 (-)
*Stenotrophomonas_maltophilia*_(CRX68037)	58.82	6.2-11.2 (-)
*Stenotrophomonas_acidaminiphila*_(ALJ29962)	57.64	7.4-12.4 (-)
Xanthomonas vesicatoria LMG919-07645 (KTF37346)	68.7	(+) 3.7-1.3 (-)
*Pantoea*_sp._CFSAN033090_(KOA70119)	58.12	6.8-11.8 (-)
*Serratia_marcescens*_(CVF42851)	58.39	6.6-11.6 (-)

The values in same colour indicates close/similar GC%

Figure A.

a)

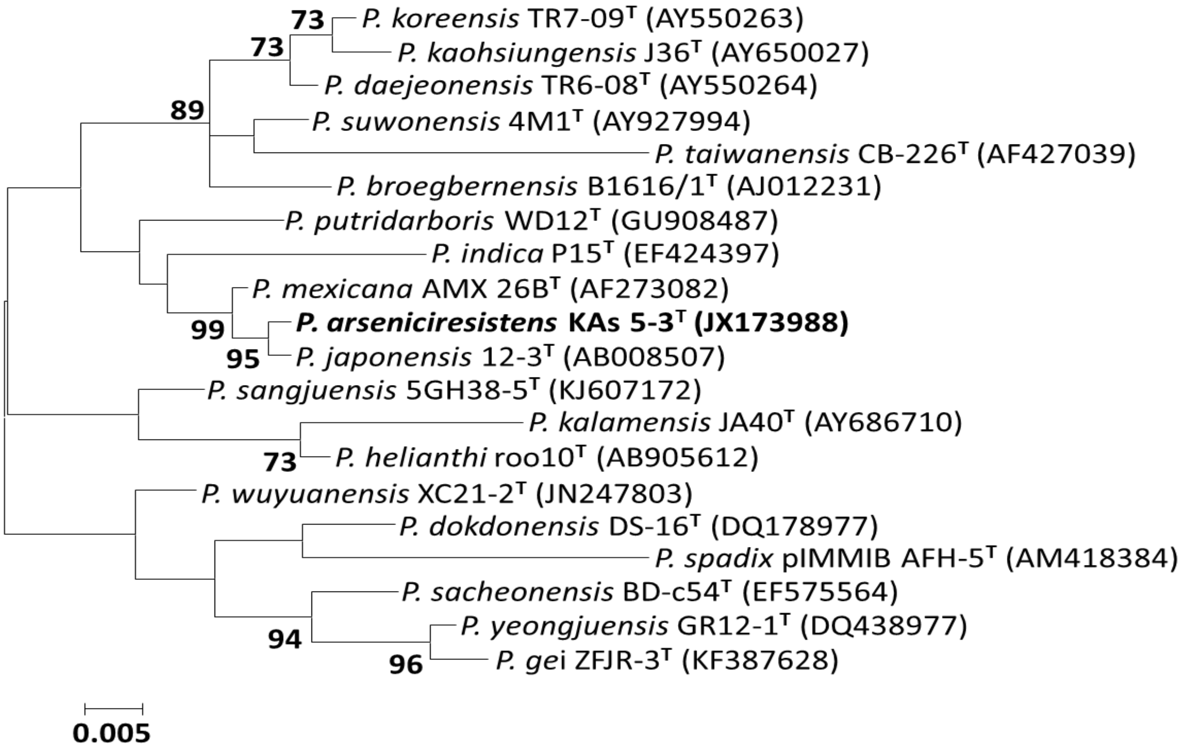

73
73
89
P. koreensis TR7-09T (AY550263)
P. kaohsiungensis J36T (AY650027)
P. daejeonensis TR6-08T (AY550264)
P. suwonensis 4M1T (AY927994)
P. taiwanensis CB-226T (AF427039)
P. broegbernensis B1616/1T (AJ012231)
P. putridarboris WD12T (GU908487)
P. indica P15T (EF424397)
P. mexicana AMX 26BT (AF273082)
P. arseniciresistens KAs 5-3T (JX173988)
P. japonensis 12-3T (AB008507)
99
95
P. sangjuensis 5GH38-5T (KJ607172)
P. kalamensis JA40T (AY686710)
P. helianthi roo10T (AB905612)
73
P. wuyuanensis XC21-2T (JN247803)
P. dokdonensis DS-16T (DQ178977)
P. spadix pIMMIB AFH-5T (AM418384)
P. sacheonensis BD-c54T (EF575564)
P. yeongjuensis GR12-1T (DQ438977)
P. gei ZFJR-3T (KF387628)
94
96
0.005

b)

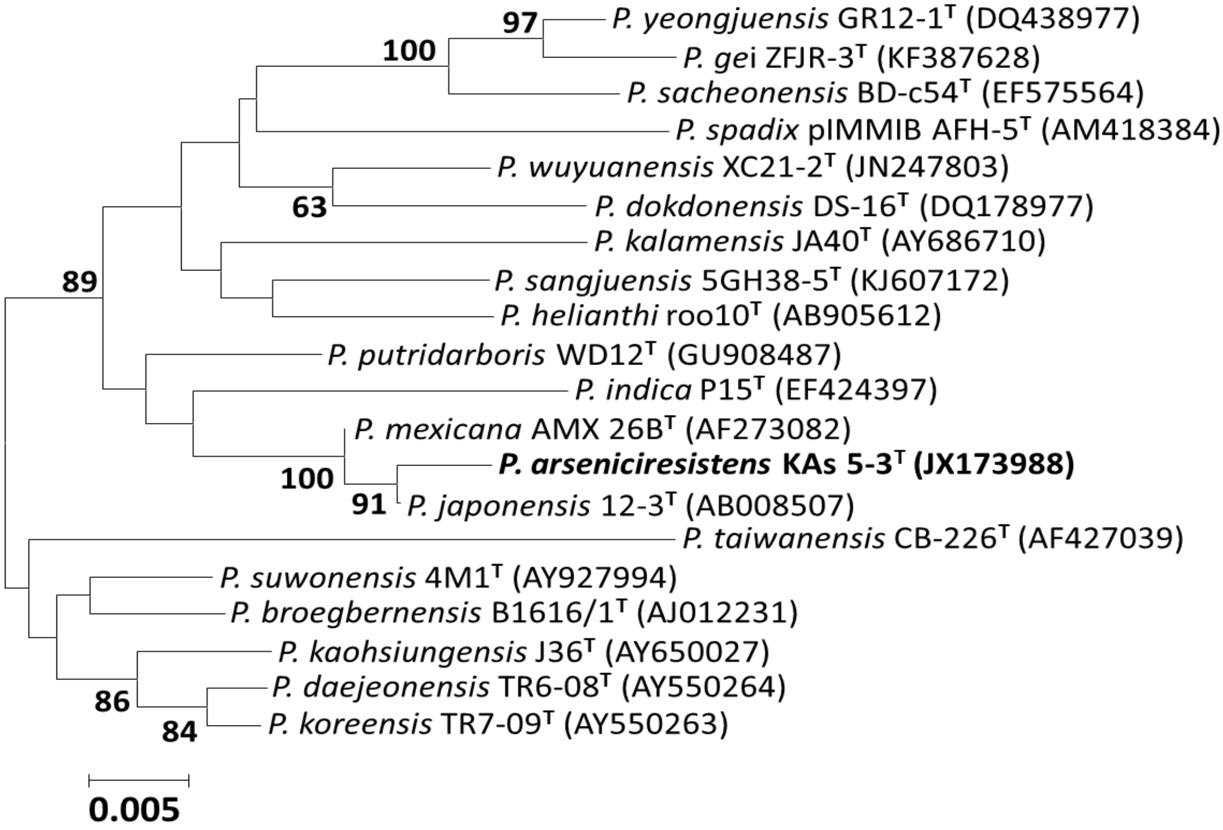

97
100
P. yeongjuensis GR12-1T (DQ438977)
P. gei ZFJR-3T (KF387628)
P. sacheonensis BD-c54T (EF575564)
P. spadix pIMMIB AFH-5T (AM418384)
P. wuyuanensis XC21-2T (JN247803)
P. dokdonensis DS-16T (DQ178977)
63
P. kalamensis JA40T (AY686710)
89
P. sangjuensis 5GH38-5T (KJ607172)
P. helianthi roo10T (AB905612)
P. putridarboris WD12T (GU908487)
P. indica P15T (EF424397)
P. mexicana AMX 26BT (AF273082)
P. arseniciresistens KAs 5-3T (JX173988)
P. japonensis 12-3T (AB008507)
100
91
P. taiwanensis CB-226T (AF427039)
P. suwonensis 4M1T (AY927994)
P. broegbernensis B1616/1T (AJ012231)
P. kaohsiungensis J36T (AY650027)
P. daejeonensis TR6-08T (AY550264)
P. koreensis TR7-09T (AY550263)
86
84
0.005

Figure B.

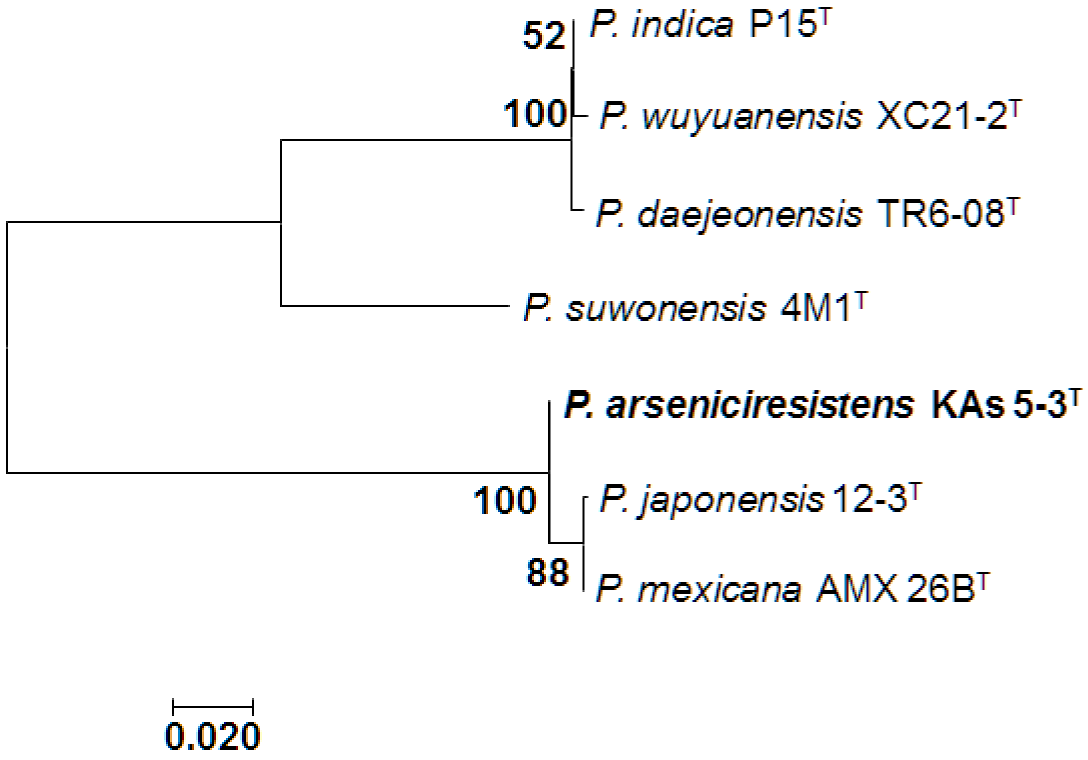

Figure C.

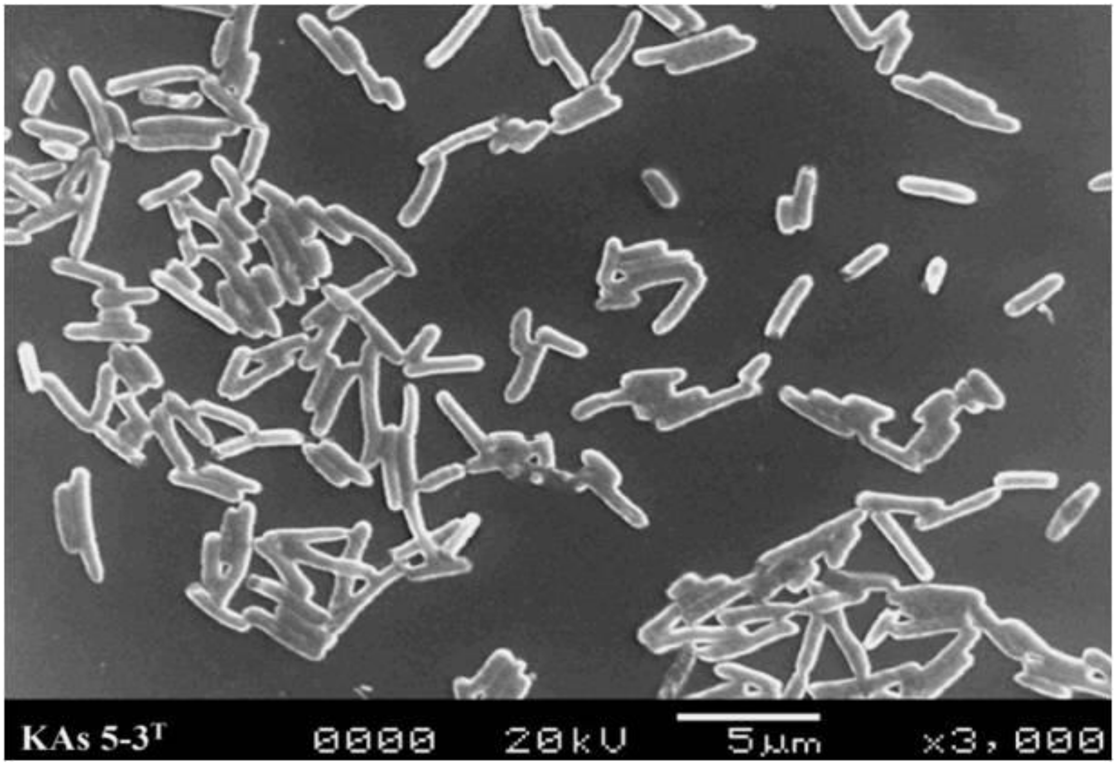

Figure D.

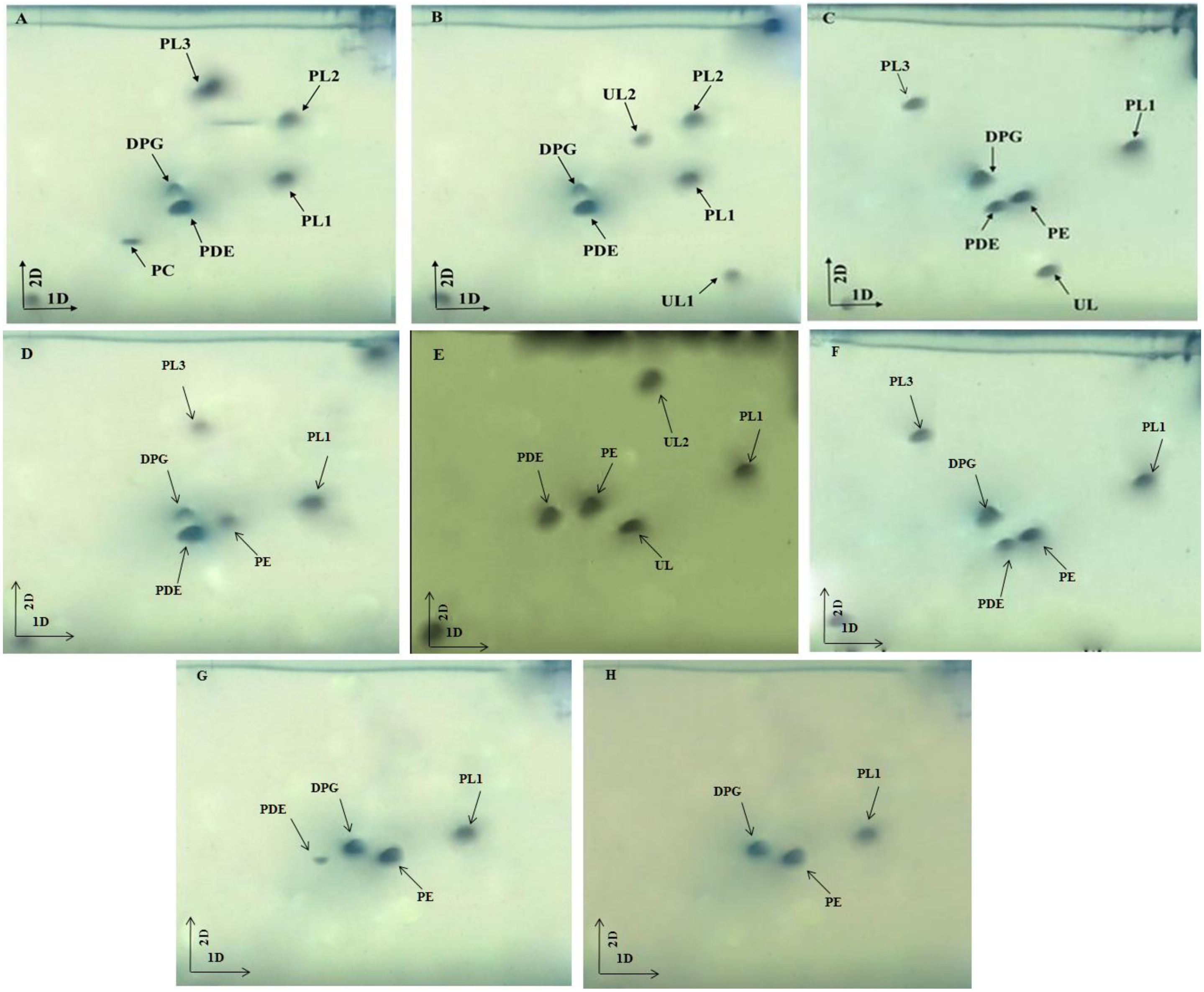
A
PL3
PL2
DPG
PL1
PDE
PC
2D
1D
B
UL2
PL2
DPG
PL1
PDE
UL1
2D
1D
C
PL3
PL1
DPG
PDE
PE
UL
2D
1D
D
PL3
PL1
DPG
PE
PDE
2D
1D
E
UL2
PL1
PDE
PE
UL
2D
1D
F
PL3
PL1
DPG
PE
PDE
2D
1D
G
DPG
PL1
PDE
PE
2D
1D
H
DPG
PL1
PE
2D
1D

Figure E. a)

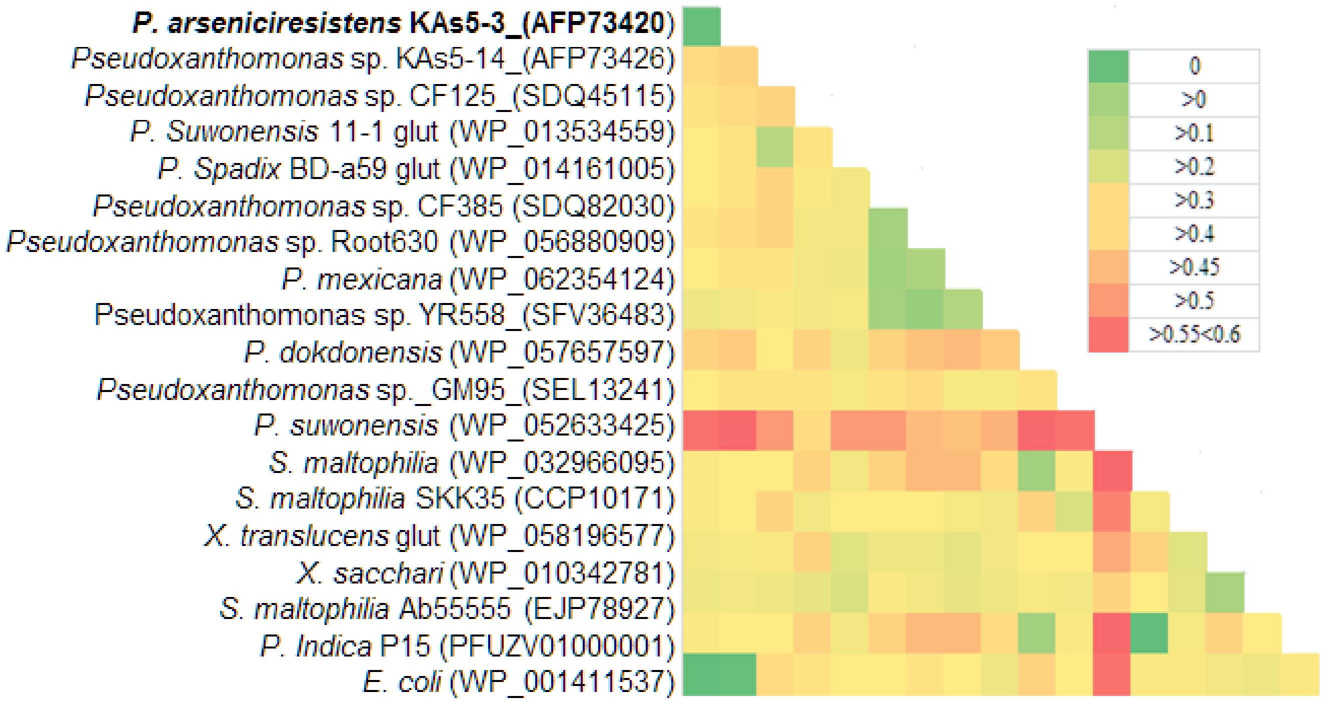
P. arseniciresistens KAs5-3_(AFP73420)
Pseudoxanthomonas sp. KAs5-14_(AFP73426)
Pseudoxanthomonas sp. CF125_(SDQ45115)
P. Suwonensis 11-1 glut (WP_013534559)
P. Spadix BD-a59 glut (WP_014161005)
Pseudoxanthomonas sp. CF385 (SDQ82030)
Pseudoxanthomonas sp. Root630 (WP_056880909)
P. mexicana (WP_062354124)
Pseudoxanthomonas sp. YR558_(SFV36483)
P. dokdonensis (WP_057657597)
Pseudoxanthomonas sp._GM95_(SEL13241)
P. suwonensis (WP_052633425)
S. maltophilia (WP_032966095)
S. maltophilia SKK35 (CCP10171)
X. translucens glut (WP_058196577)
X. sacchari (WP_010342781)
S. maltophilia Ab55555 (EJP78927)
P. Indica P15 (PFUZV01000001)
E. coli (WP_001411537)
0
>0
>0.1
>0.2
>0.3
>0.4
>0.45
>0.5
>0.55<0.6

b)

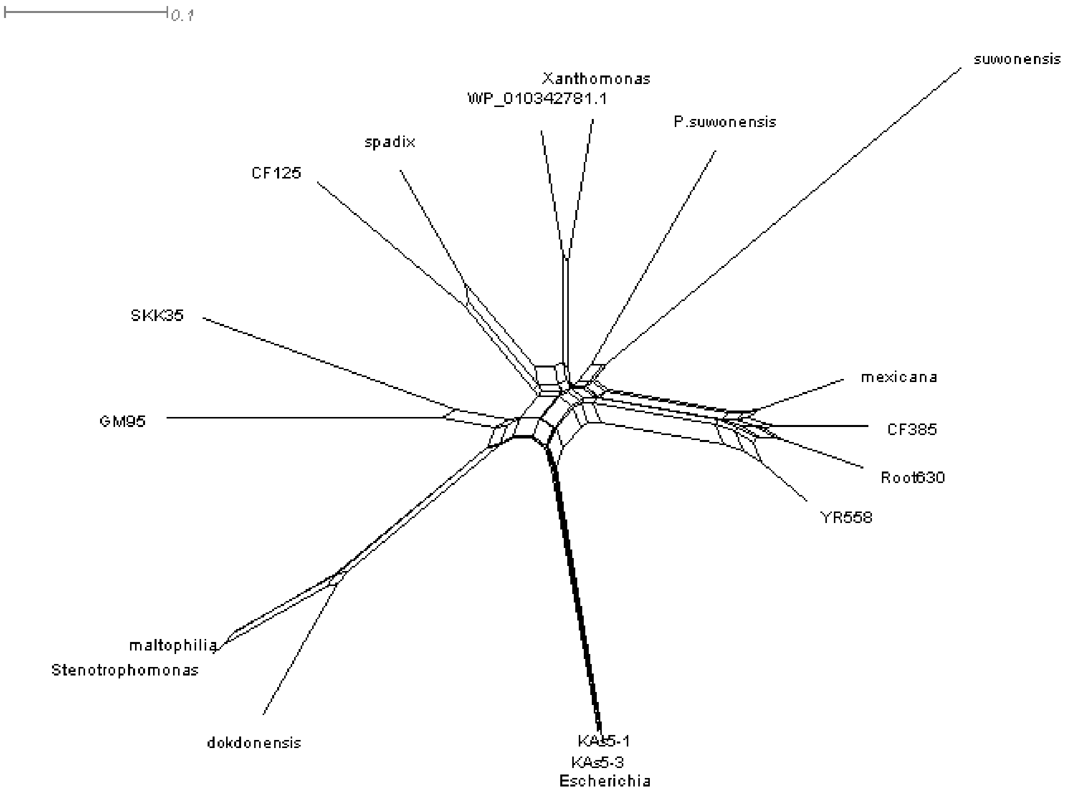
0.1
suwonensis
Xanthomonas
WP_010342781.1
P.suwonensis
spadix
CF125
SKK35
GM95
mexicana
CF385
Root630
YR558
maltophilia
Stenotrophomonas
dokdonensis
KAs5-1
KAs5-3
Escherichia

Figure F. a)

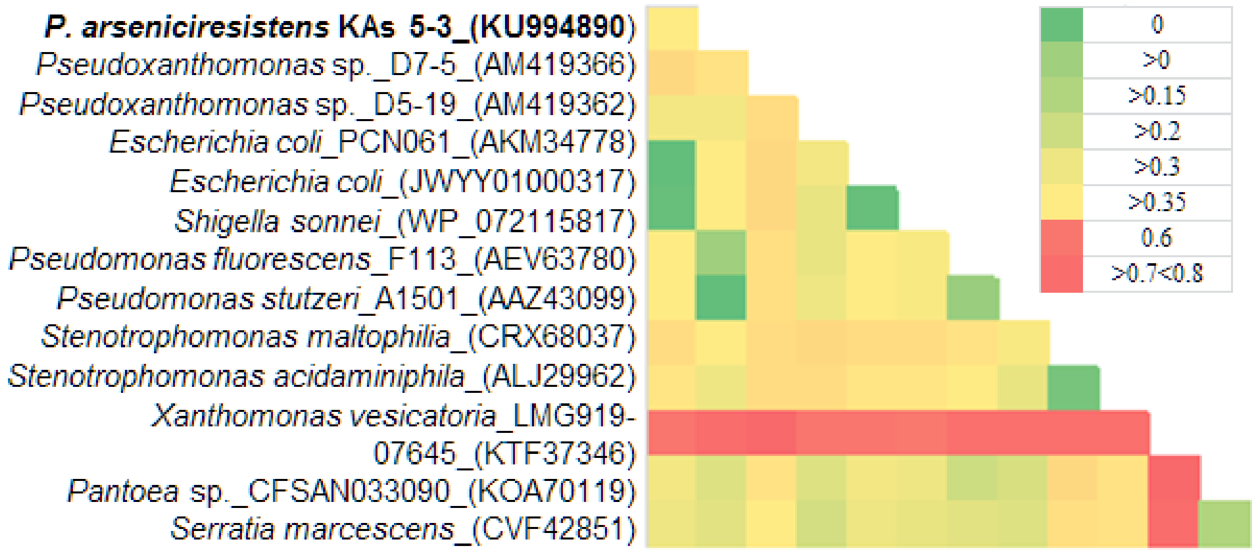
P. arseniciresistens KAs 5-3_(KU994890)
Pseudoxanthomonas sp._D7-5_(AM419366)
Pseudoxanthomonas sp._D5-19_(AM419362)
Escherichia coli_PCN061_(AKM34778)
Escherichia coli_(JWYY01000317)
Shigella sonnei_(WP_072115817)
Pseudomonas fluorescens_F113_(AEV63780)
Pseudomonas stutzeri_A1501_(AAZ43099)
Stenotrophomonas maltophilia_(CRX68037)
Stenotrophomonas acidaminiphila_(ALJ29962)
Xanthomonas vesicatoria_LMG919-07645_(KTF37346)
Pantoea sp._CFSAN033090_(KOA70119)
Serratia marcescens_(CVF42851)
0
>0
>0.15
>0.2
>0.3
>0.35
0.6
>0.7<0.8

b)

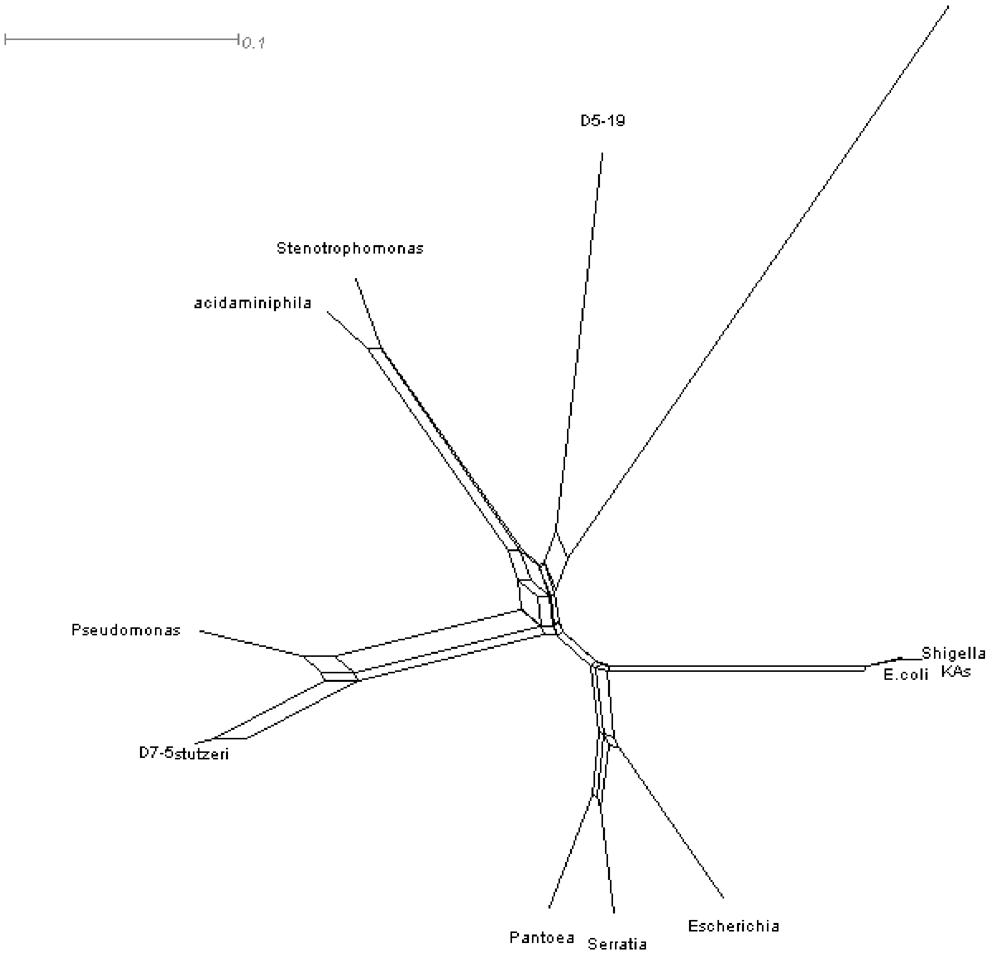
0.1
D5-19
Stenotrophomonas
acidaminiphila
Pseudomonas
D7-5
stutzeri
Shigella
E.coli
KAs
Pantoea
Serratia
Escherichia
