# Supplementary material for: Taxonomy and physiology of Pseudoxanthomonas arseniciresistens sp. nov., an arsenate and nitrate-reducing novel gammaproteobacterium from arsenic contaminated groundwater, India
Source: PLoS One. 2018 Mar 20;13(3):e0193718. doi: 10.1371/journal.pone.0193718 (PMC5860741; doi:10.1371/journal.pone.0193718)
Supplement: S1 Certificate — A, Microbial culture collection (MCC) deposition certificate of strain KAs 5-3T. B, Microbial type culture collection (MTCC) deposition certificate of strain KAs 5-3T. C, Belgian coordinated culture collection (BCCM) deposition certificate of strain KAs 5-3T. (PDF) [file pone.0193718.s002.pdf]

MCC

सुक्ष्मजीव संवर्धन संकलन

MICROBIAL CULTURE COLLECTION

Member of WFCC, An IDA under Budapest Treaty, DNR of MoEF

राष्ट्रीय कोशिका विज्ञान केंद्र | National Centre for Cell Science

Certificate Of Deposit

***Pseudoxanthomonas* sp.** strain **KAs 5-3**

was received for public access deposit at MCC from

Dr. Pinaki Sar

Department of Biotechnology, Indian Institute of Technology

Kharagpur - 721302, West Bengal

India

on **29 February 2016**

and was allocated the accession number

MCC 3121

After confirming the viability, purity and authenticity of the strain in the MCC facilities, it has been preserved using standard methods.

The strain is now available to the public.

Kamlesh

Dr. Kamlesh Jangid

Scientist, Microbial Culture Collection, Pune

11 August 2016

साई ट्रिनिटी कॉम्प्लेक्स, सुस रोड, पाषाण, पुणे- . महाराष्ट्र, भारत.

Sai Trinity Complex, Sus Road, Pashan, Pune- 411 021. Maharashtra, INDIA

दुरभाष / Tel: +9120 25329000 | फ़ैक्स / Fax: +9120 25329001

ई-मेल / e-mail: mcc@nccs.res.in | वेब / Web: www.nccs.res.in/mcc

एनसीसीएस भारत सरकार के जैवप्रौद्योगिकी विभाग का स्वायत्त संस्थान है.

NCCS is an Autonomous Institute of Department of Biotechnology, Govt. of India

Registered Post

MTCC

MICROBIAL TYPE CULTURE COLLECTION & GENE BANK

सैक्टर 39 ए, चण्डीगढ़ 160036, भारत

INSTITUTE OF MICROBIAL TECHNOLOGY

(A CONSTITUENT ESTABLISHMENT OF CSIR)

Sector 39-A, Chandigarh-160036 (INDIA)

IMTECH

Dr. Suresh Korpole
Scientist

Date: 02-09-2014

To,

Dr. Pinaki Sar
Associate Professor
Department of Biotechnology
Indian Institute of Technology
Kharagpur -731302, INDIA

Dear Dr. Sar,

Please find enclosed the MTCC accession numbers given to your strains *Acinetobacter* sp. BAS 123i (12113), *Staphylococcus* sp. BAS 106i (12114), *Rhizobium* sp. KAS5-22 (12115), *Pseudoxanthomonas* sp. KAs 5-3(12116), *Achromobacter* sp. WKs - 3-5 (12117), *Bacillus p. lII J3 (12118)*submitted for general deposition. The **16S** rRNA gene sequencing showed more than 99 % match with the sequences provided by you.

Thanking you for your interest in MTCC.

Best regards.
Yours sincerely,

(K. Suresh)

Phones : +91-172-2690562
2636680-94

Fax : +91-172-2695215
2690632
2690585

E-mail : curator@imtech.res.in
Web : http://mtce.imtech.res.in

GREAT AT SMALL THINGS

BCCM DCG

BCCM IHEM

BCCM ITM

BCCM LMBP

BCCM MUCL

BCCM ULC

BCCM CC

CERTIFICATE OF DEPOSIT

This is to certify that the following microorganism has been deposited into the BCCM/LMG Bacteria Collection and is available to the public without restriction:

LMG number: **LMG 29169**

Speciesname: **Pseudoxanthomonas sp.**

Depositor: **Sar Pinaki, Indian Institute of Technology (IIT) - Kharagpur, Dept. of Biotechnology, Environmental Microbiology Lab**

Depositor no: **KAs 5-3**

Gent, 01 February 2016

ir. Claudine Vereecke
Public Collection Curator
BCCM/LMG Bacteria Collection

Laboratorium voor Microbiologie - Universiteit Gent (UGent)
K.L. Ledeganckstraat 35 - B-9000 Gent - Belgium
T +32 (0)9 264 51 08 - bccm.lmg@ugent.be
